# Transcription factor Foxo1 is essential for IL-9 induction in T helper cells

**DOI:** 10.1038/s41467-017-00674-6

**Published:** 2017-10-09

**Authors:** Sakshi Malik, Srikanth Sadhu, Srikanth Elesela, Ramendra Pati Pandey, Amanpreet Singh Chawla, Deepak Sharma, Lipsa Panda, Deepak Rathore, Balram Ghosh, Vineet Ahuja, Amit Awasthi

**Affiliations:** 1 Center for Human Microbial Ecology, Translational Health Science & Technology Institute, NCR Biotech Science Cluster, 3rd Milestone Gurgaon-Faridabad Expressway, Faridabad, Haryana 121 001 India; 2 National Institute of Immunology, Aruna Asaf Ali Marg, New Delhi, 110067 India; 3 Department of Biotechnology, Indian Institute of Technology Roorkee, Roorkee, 247667 India; 4 Institute of Genomics and Integrative Biology (IGIB), Mall Road, New Delhi, 110007 India; 5 Department of Gastroenterology, All India Institute of Medical Sciences (AIIMS), Ansari Nagar, New Delhi, 110029 India

## Abstract

Interleukin 9 (IL-9)-producing helper T (Th9) cells have a crucial function in allergic inflammation, autoimmunity, immunity to extracellular pathogens and anti-tumor immune responses. In addition to Th9, Th2, Th17 and Foxp3^+^ regulatory T (Treg) cells produce IL-9. A transcription factor that is critical for IL-9 induction in Th2, Th9 and Th17 cells has not been identified. Here we show that the forkhead family transcription factor Foxo1 is required for IL-9 induction in Th9 and Th17 cells. We further show that inhibition of AKT enhances IL-9 induction in Th9 cells while it reciprocally regulates IL-9 and IL-17 in Th17 cells via Foxo1. Mechanistically, Foxo1 binds and transactivates IL-9 and IRF4 promoters in Th9, Th17 and iTreg cells. Furthermore, loss of Foxo1 attenuates IL-9 in mouse and human Th9 and Th17 cells, and ameliorates allergic inflammation in asthma. Our findings thus identify that Foxo1 is essential for IL-9 induction in Th9 and Th17 cells.

## Abstract (web summary)

The transcription factor Foxo1 can control regulatory T cell and Th1 function. Here the authors show that Foxo1 is also critical for IL-9 production by Th9 cells and other IL-9-producing cells.

## Introduction

Interleukin 9 (IL-9), a pleiotropic cytokine of common γ-chain cytokine receptor family, has a crucial function in allergic inflammation, autoimmunity, immunity to extracellular pathogens^1^ and anti-tumor immunity^2, 3^. IL-9 secretion was initially shown to be associated with T helper (Th) 2 cells in Th2-associated infection and allergic inflammation models. Although Th2, Th17 and Foxp3^+^ regulatory T (Treg) cells produce IL-9^4–8^, Th9 cells are a more specialized IL-9-producing cell and have been shown to be proinflammatory in vivo^9, 10^.

Antigenic stimulation of naive CD4^+^ T cells together with transforming growth factor-β (TGF-β) and IL-4 can induce the developmental program of Th9 cells. IL-4 restrains the development of TGF-β-induced Foxp3^+^ T (iTreg) cells by suppressing Foxp3 expression and reprograms them into IL-9-producing Th9 cells^9, 10^. Similar to mice Th9 cells, human Th9 cells are implicated in the development of allergic and autoimmune diseases^5^.

Despite seminal work on the differentiation and development of Th9 cells, the transcriptional program controlling development of Th9 cells and IL-9-producing T cells is not clear. Although IRF-4, PU.1, BATF and IRF-1 are critical for inducing IL-9 in Th9 cells^3, 11–13^, these transcription factors are also essential for the differentiation of other effector Th lineages as well as B cell development. IRF-4 and BATF have been suggested to be required for the development of Th17 cells^14, 15^. PU.1 was shown to promote the development of B cells and macrophages, and IRF1 has shown to be essential for development and functions of Th1 cells^16^, Taken together it clearly suggests that a distinct transcription factor is required for the development of Th9 and IL-9-producing T cells.

In addition to Th9 cells, Th17 cells produce IL-9, which is suppressed by IL-23^6, 17^. Interestingly, IL-23 controls the balance between IL-9 and IL-17 induction by suppressing or enhancing their expression in Th17 cells^17, 18^. Although, multiple mechanisms have been suggested by which IL-23 enhances IL-17 expression and the Th17 phenotype, the underlying mechanism of IL-23-mediated suppression of IL-9 expression in Th17 cells is not clearly understood. IL-23-mediated regulation of Foxo1 activity has been shown to enhance the development and effector functions of Th17 cells^18^. Another study demonstrated that a T cell-intrinsic deletion of Foxo1 increases Th17 development and function via enhancing RoRγt functions, as Foxo1 suppresses RoRγt activity^19^.

Foxo1, a member of forkhead box O (Foxo) family that includes Foxo3 and Foxo4, regulates various cellular processes, including cell survival, apoptosis and Th cell differentiation^20^. Foxo1 and Foxo3 are highly expressed in Foxp3^+^ Treg cells^21, 22^, and Foxp3-dependent deletion of Foxo1 in Treg cells impairs Treg cell generation and suppressive functions^21, 23^. Moreover, Foxo1-deficient Treg cells produce more IFN-γ as compared to wild-type (Wt) Treg cells, and this distinction can mediate colitis pathology^23^. Similarly, Foxo1 can negatively regulate the generation of Th1 cells by suppressing T-bet function^21, 24^. However, the role of Foxo1 in the development of Th9 cells has not been addressed.

The functions of Foxo1 are regulated transcriptionally and post-transcriptionally. The post-transcriptional functions of Foxo1 are regulated by its phosphorylation and acetylation^25^. The activation or inactivation of transcriptional activity induced by Foxo1 is tightly controlled by its upstream kinases, SGK1 and AKT^18^. AKT-mediated phosphorylation of Foxo1 at Thr24, Ser256 and Ser319 inactivates its transcriptional activity^25, 26^. Although Foxo1 activity is primarily measured post-transcriptionally, its activity can also be detected at the mRNA level, as transcriptionally active Foxo1 induces its own expression^27^.

Stimulation of T cells with antigen activates the PI(3)K/AKT pathway, which drives effective T cell responses. Activated AKT phosphorylates Foxo1 within the nucleus to induce its relocalization from nucleus to cytosol, and thereby inactivates its transcriptional activity. Although the role of AKT-Foxo1 axis has been described in Th17 and Treg cells, such functions have not been identified in IL-9 induction in Th17 and Th9 cells.

Here we show that Foxo1 is differentially expressed in Th9 cells, and is required for the induction of IL-9 in Th2, Th9, Th17 and iTreg cells. We further identify that AKT negatively regulates IL-9 induction in Th9 and Th17 cells by inhibiting Foxo1 functions. Foxo1 physically binds and transactivates the IL-9 locus in Th9, Th17 and iTreg cells. Foxo1 also binds and transactivates the IRF4 locus, which is essential for the development of Th9 cells. Furthermore, loss of Foxo1 suppresses IL-9 production in mouse and human Th9 and Th17 cells and substantially ameliorates allergic inflammation in asthma. Our findings thus identify Foxo1 as a major transcription factor controlling the development of Th9 cells and other IL-9-producing T cells.

## Results

### Foxo1 is differentially expressed in Th9 cells

**Fig. 1:**
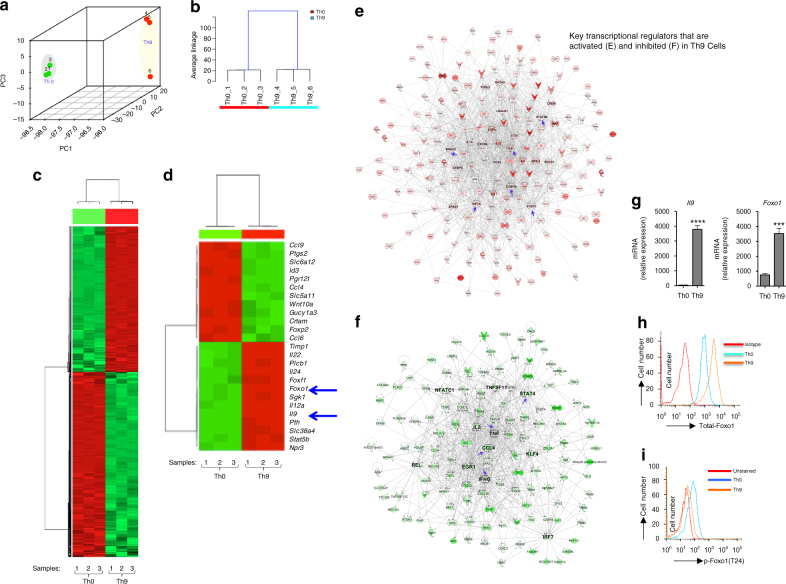
Identification of Foxo1 in differentiated Th9 cells. Naive CD4^+^CD62L^+^CD44^−^ T cells were isolated from wild- type (Wt) C57/BL6 mice were and then differentiated into helper T (Th) 9 (TGF-β1 + IL-4) or Th0 (without skewing cytokines) conditions. Samples were collected for RNASeq analysis at 72 h time point. Unsupervised analysis of Th9 and Th0 cells transcriptome data, **a** PCA analysis and **b** Clustering analysis. Significantly differentially expressed genes between Th9 and Th0, **c** heat-map of all significantly differentially expressed genes, **d** heat-map analysis of selected top significantly differentially expressed genes. System biology analysis of the key transcriptional regulators that are activated **e** and inhibited **f** in Th9 Cells as compared to Th0 cells. **g** qPCR analysis of *Il9* and *Foxo1* in Th9 cells induced by TGF-β1 + IL-4, results were normalized to the expression of mouse *Gapdh*, and are presented relative to those of control Th0 cells. *Bar* shows mean ± s.d. from combined three experiments. ***P < 0.001. ****P < 0.0001 (unpaired student *t-*test). Flow cytometry analysis of total **h** and phospho Foxo1 **i**﻿ in Th0 and Th9 cells, data representative of two independent experiments

We have previously reported that TGF-β1 together with Interleukin (IL-) 4 induces the differentiation of IL-9-producing helper T (Th) 9 cells^9^. In addition to Th9 cells, Th2 and Th17 cells also produce IL-9 albeit at lower levels. Although transcription factors like IRF-4, PU.1, BATF and IRF-1 are shown to be critical for IL-9 induction in Th9 cells^3, 11–13^, transcriptional regulation of IL-9 in other Th cells is not clearly understood. To understand and obtain the detailed transcriptional program induced in naive CD4^+^ T cells (Supplementary Fig. 1a, b) differentiated into Th9 cells by TGF-β1 plus IL-4, we analyzed the global gene expression profile following engagement of TCR on naive CD4^+^ T cells cultured in the presence of Th9 polarizing cytokines (TGF-β1 plus IL-4) and compared this gene expression profile with T cell activated in the absence of any polarizing cytokines (Th0). This experimental design allows us to identify the transcription factors essential for the development of Th9 cells. Unsupervised PCA analysis indicated that Th9 and Th0 cells are two different cell types although they originate from same naive CD4^+^ T cell precursors (Fig. 1a, b). System biology analysis identified the significantly differentially expressed genes in Th9 as compared to Th0 cells (Fig. 1c). Our bioinformatics analysis further identified top significantly expressed genes of Th9 cells, and interestingly Foxo1 was identified amongst highly ranked putative transcription factor expressed in Th9 as compared to Th0 cells (Fig. 1c, d). In addition, our system biology analysis identified the key transcriptional regulators that are activated and inhibited in Th9 as compared to Th0 cells (Fig. 1e, f). Our analysis has confirmed the upregulation of known Th9-associated cytokine transcripts, *Il9*, *Il21* and *Il10* (Fig. 1e). In addition, our global gene profiling of Th9 cells also confirmed the upregulation of the transcripts of known Th9-associated transcription factors such as *Samd3*, *Gata3*, *Batf3*, *Stat5b* and *Hif1a* ^13, 28–30^ (Fig. 1e). To further corroborated our findings of the identification of Foxo1 in Th9 cells, we also reanalyzed published microarray data that were set to identify Th9 exclusive-gene signature as compared to Th2 cells, as both of these cell types are closely related due to their shared differentiation factor^13^. Among the top 250 differentially expressed genes, *Foxo1* is substantially upregulated in Th9 as compared to Th2 cells (Supplementary Data 1 and Supplementary Fig. 2a).

To further confirm expression of Foxo1 during Th9 differentiation, we first validated the mRNA expression of Foxo1 using qPCR (quantitative polymerase chain reaction). In agreement with RNAseq data, *Foxo1* is differentially expressed in Th9 as compared to Th0 cells (Fig. 1g, *right panel*). We further confirmed that as compared to Th0, Th9 cells express higher levels of total Foxo1 protein (Fig. 1h). Since the functions of Foxo1 are regulated by its phosphorylation, therefore we tested phosphorylation status of Foxo1 in Th9 cells. We found that phosphorylation of Foxo1 was decreased in Th9 cells as compared to Th0 cells (Fig. 1i). We further performed time kinetics analysis of *Foxo1* and *Il9* mRNA expression in Th9 cells. We found that *Foxo1* mRNA was detected as early as 60 min and remained upregulated after 12 h while *Il9* mRNA expression was detected after 12 h and remained upregulated at later time points in Th9 cells (Supplementary Fig. 2b, c). Taken together these observations indicated that *Foxo1* is differentially expressed and might play a role in Th9 cells development.

### Th9-enhancing factors increased Foxo1 in Th9 cells

**Fig. 2:**
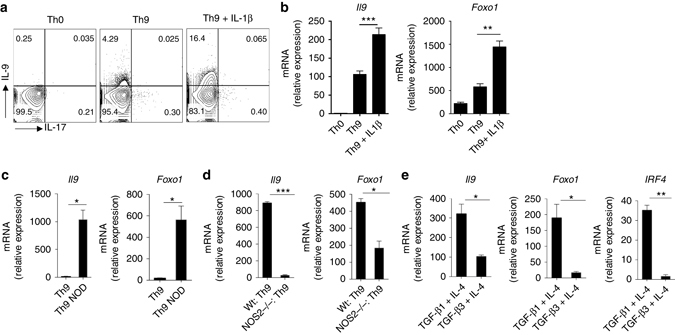
IL-1β and nitric oxide induces Foxo1 in Th9 cells. **a**–**c** Naive CD4^+^CD62L^+^CD44^−^ T cells were isolated from WT mice and then treated under Th9 (TGF-β1 + IL-4) or Th0 (without skewing cytokines) conditions with/without IL-1β or nitric oxide donor (NOC-18, 50 μM) as indicated. **a** After 72 h, cells were restimulated and then intracellular expression of IL-9 and IL-17 was determined by flow cytometry. **b**, **c** After 72 h, cells were harvested, RNA was prepared and qPCR was performed for determining the expression of *Il9* and *Foxo1*. **d** Wt and *Nos2−/−* naive CD4^+^ T cells were isolated and cultured into Th9 condition, qPCR was performed for the expression of *Il9* and *Foxo1*. **e** Naive CD4^+^ T cells were stimulated and polarized with TGF-β1 + IL-4 or with TGF-β3 + IL-4, *Il9*, *Foxo1* and *Irf4* mRNA was determined by qPCR. Contour plot is representative of two experiments **a**. *Bar* shows mean ± s.d. from combined three **b**–**e** experiments. *P < 0.05 **P < 0.01, ***P < 0.001 (Two-way variance analysis (analysis of variance))

Similar to other Th cells, the generation of Th9 cells can be further enhanced. OX40, TSLP, IL-1β and nitric oxide were shown to enhance the generation of Th9 cells induced by TGF-β1 plus IL-4. However, it is not yet identified whether Th9-enhancing factors can also increase the Foxo1 expression in Th9 cells. To test this, we have generated Th9 cells in the presence or absence of IL-1β and nitric oxide. Interestingly, Th9-enhancing factor, IL-1β^3, 31^ enhanced the expression of *Il9* and *Foxo1* in Th9 cells (Fig. 2a, b). In addition to IL-1β, nitric oxide^32^ also induced the enhanced expression of *Il9* and *Foxo1* in Th9 cells (Fig. 2c). On contrary, as compared to wild-type (Wt), *N*os2^−/−^ Th9 cells failed to induce *Il9* and *Foxo1* expression (Fig. 2d). We have earlier shown that TGF-β3, instead of TGF-β1, induced pathogenic Th17 cells express lower levels of IL-9^17^. We wanted to test whether TGF-β3-induced Th17 cells express lower level of Foxo1 as compared to TGF-β1-induced Th17 cells. To do this, we first reanalyzed published microarray data of TGF-β1- and TGF-β3-induced Th17 cells^17^, and found that both *Il9* and *Foxo1* are highly expressed in TGF-β1, but not in TGF-β3, induced Th17 cells (Supplementary Fig. 2d). Since TGF-β1 and TGF-β3 differentially regulate IL-9 and Foxo1 in Th17 cells, we wanted to test whether TGF-β1 or TGF-β3 together with IL-4 induces differentially induce Th9 cells differentiation and Foxo1 expression. To do this, we differentiated Th9 cells in the presence of TGF-β1 or TGF-β3 plus IL-4. qPCR analysis confirmed that as compared to TGF-β1, TGF-β3 together with IL-4 induced attenuated expression of *Il9* and *Foxo1* (Fig. 2e). All together, these data demonstrated an association of Foxo1 with IL-9 in Th9 cells.

### PI(3)K/AKT regulates IL-9 induction in Th9 cells

**Fig. 3:**
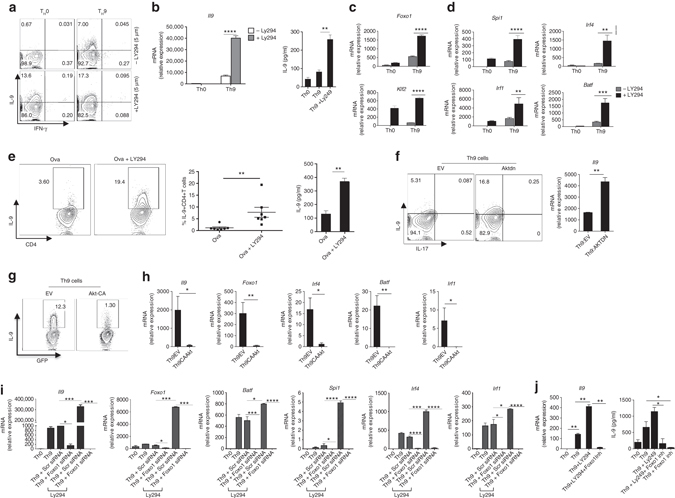
AKT-Foxo1 axis regulates the induction of IL-9 in Th9 cells. **a**–**d** Naive CD4^+^CD62L^+^CD44^−^ T cells were isolated from Wt mice and then treated under Th9 (TGF-β1 + IL-4) or Th0 (without skewing cytokines) conditions with/without LY294002 (5–10 μM) as indicated. **a** At 72 h, cells were restimulated, then intracellular expression of IL-9 and IFN-γ was determined by flow cytometry. **b** At 72 h, ELISA and qPCR for IL-9 was performed. **c**, **d** qPCR was performed for determining mRNA expression of *Foxo1*, *Irf4*, *Spi1*, *Batf*, *Irf1* and *Klf2*. Contour plot is representative of two independent experiments. *Bar* shows mean + s.d. from combined three **b**–**d**. **P < 0.01 ***P < 0.001, ****P < 0.0001 (Two-way variance analysis (analysis of variance) and Student’s *t*-test). **e** Balb/c mice were immunized with ova and treated with LY294002, 6–8 days later, spleen and LN cells were restimulated by OVA peptide (*n* = 7), % of IL-9^+^CD4^+^ T cells and IL-9 concentration were determined by flow cytometry and ELISA. Mean + s.e.m. **P < 0.01 (Student’s *t*-test). **f**–**h**, Naive CD4^+^ T cells were differentiated into Th9 cells and then retrovirally transduced with GFP empty virus or AKT-DN-GFP (dominant-negative AKT) or CA-AKT-GFP (constitutive active AKT). After 72 h, cells were restimulated and then intracellular expression of IL-9 and IL-17 was determined by flow cytometry gated on GFP^+^ cells **f**. mRNA expression of *Il9* was quantified by quantitative PCR (qPCR) **f**. **g** percentage of GFP^+^IL-9^+^ CD4^+^T cells, data representative of two different experiments. **h** mRNA expression of *Il9, Foxo1, Irf4, Batf, Irf1* mRNA in GFP sorted retrovirally transduced Th9 cells. **i** Naive CD4^+^T cells were stimulated in Th9 conditions and transfected with Scr siRNA or Foxo1 siRNA with and without LY294002 (5 μM). *Il9*, *Foxo1*, *Pu.1*, *Batf*, *Irf4*, *Irf1* mRNA was assessed by qPCR. **j** Naive CD4^+^T cells were differentiated into Th0 and Th9 with and without LY294002 (5.0 μM) and Foxo1 inhibitor (25 nM), *Il9* mRNA was quantified by qPCR and IL-9 concentration in culture supernatants was estimated by ELISA. *Bars* show mean ± s.d. from combined three **i**, **j**, two **f**, **h** experiments. *P < 0.05, **P < 0.01, ***P < 0.001 (Student’s *t*-test)

While transcriptionally active Foxo1 induces its own expression, AKT-mediated Foxo1-phosphorylation at Thr24, Ser256 and Ser319 inactivates its transcriptional activity^25, 26^. We enhanced Foxo1 transcriptional activity by inhibiting PI3K/AKT in Th9 cells, and found that PI(3)K/AKT inhibition significantly enhanced *Il9* mRNA expression as well as IL-9 protein in Th9 cells (Fig. 3a, b). Furthermore, inhibition of PI(3)K/AKT also enhanced the expression of *Foxo1* and *Klf2*, a direct Foxo1 target gene, as well as the transcription factors, *Irf4, Irf1, Spi1* and *Batf*, that are involved in Th9 development (Fig. 3c, d). In addition, inhibition of PI3K/AKT in Th9 cells suppressed phosphorylation of Foxo1 (Supplementary Fig. 3a). Dose-dependent PI(3)K/AKT-inhibition enhanced *Il9* expression in Th9 cells (Supplementary Fig. 3b, c). In fact, PI(3)K/AKT-inhibition induced expression of *Il9* and Th9-associated genes (*Irf4, Irf1, Gata3, Batf and Spi1*) even in un-polarized activated T cells (Supplementary Fig. 3d). Consistently, in vivo inhibition of PI(3)K/AKT enhanced the frequency ova-specific IL-9^+^CD4^+^ T cells and IL-9 production upon immunization (Fig. 3e). While dominant-negative AKT (AKTdn) enhanced *Il9* expression and inhibited AKT and Foxo1 phosphorylation (Fig. 3f and Supplementary Fig. 3e), constitutive-active AKT (AKT-CA) suppressed IL-9, *Foxo1* and Th9-associated genes (*Irf4*, *Batf* and *Irf1*) in Th9 cells (Fig. 3g, h). In addition, AKT-CA overexpression increased phosphorylation of Foxo1 (Supplementary Fig. 3f), which might control Foxo1 functions.

Foxo1-inhibition by siRNA reversed the effects of PI(3)K/AKT-inhibition on *Il9* in Th9 and Tc9 cells as well as known Th9-associated transcription factors, *Irf4*, *Batf*, *Spi1* and *Irf1* in Th9 cells (Fig. 3i and Supplementary Fig. 4a, b). In addition, Foxo1-specific inhibitor (AS1842856) suppressed the effect of PI(3)K/AKT-inhibition on IL-9 induction (Fig. 3j), suggesting that AKT-inhibition enhances IL-9 via Foxo1 in Th9 cells. All together, these results clearly demonstrated PI(3)K/AKT-mediated regulation of Th9 cells development.

### Foxo1 reciprocally regulates IL-17 and IL-9 in Th17 cells

**Fig. 4:**
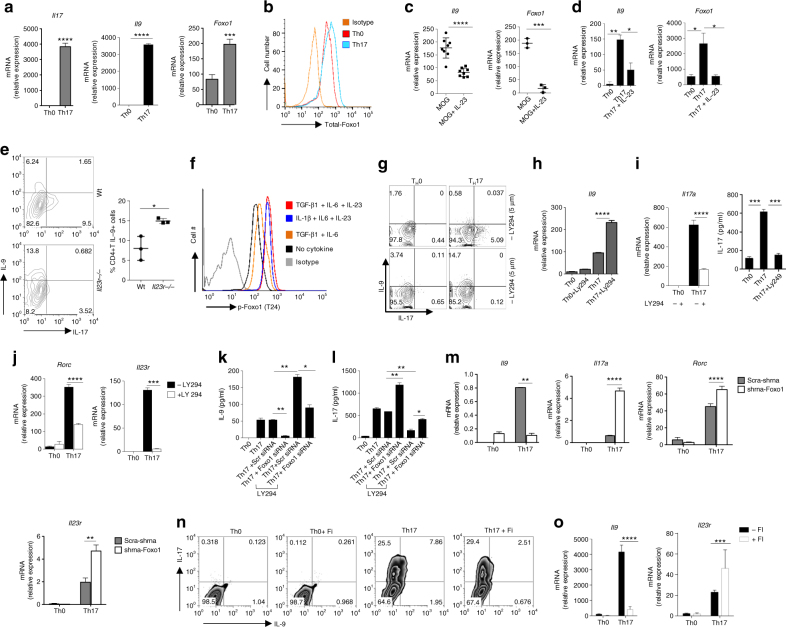
Foxo1 reciprocally regulates IL-17 and IL-9 in Th17 cells. **a**, **b** Naive T cells were differentiated into Th0 and Th17 cells, *Il17*, *Il9*, *Foxo1* mRNA was determined by qPCR **a**, total Foxo1 was determined by FACS, data representative of at least two independent experiments **b**. **c** Wt B6 mice were immunized with MOG_35–55,_ 8 day later, cells were restimulated with MOG_35–55_ with or without IL-23, mRNA of *Il9* (*n* = 8) and *Foxo1* (*n* = 3) was determined by qPCR. **d** Naive T cells were differentiated into Th17 cells with or with IL-23, at 72 h mRNA expression of *Il9* and *Foxo1* was determined. **e** Wt and *Il23r−/−* mice were immunized with MOG_35–55_ (*n* = 3), cells were restimulated and then IL-9/IL-17 production was determined by FACS, bars to the right given percentage of IL-9^+^CD4^+^ T cells. **f** Flow cytometric analysis of pFoxo1 in T cells treated with indicated culture conditions, data represent of two independent experiments. **g**–**j** Naive T cells were differentiated into Th0 and Th17 conditions with or without LY294002 (5.0 μM). IL-9 and IL-17 production was determined by flow cytometry **g**, mRNA expression of *Il9*, *Il17a*, *Rorc* and *Il23r* was determined by qPCR **h**–**j**, IL-17 concentration was determined by ELISA from culture supernatant (**i**, *right panel*). Contour plot is representative of two experiments **g**. **k**, **l** Naive T cells were differentiated in Th0 and Th17 condition, transfected with Scr-siRNA or Foxo1-siRNA and treated with or without LY294002 as indicated. At 72 hr, IL-9 and IL-17 concentrations was determined by ELISA. **m** qPCR analysis of *Il9*, *Il17*, *Rorc*, *Il23r* from Th17 cells transduced with Scr-shRNA or Foxo1-shRNA. **n** In vitro differentiated Th17 cells were treated with or without Foxo1 inhibitor (25 nM), and IL-9/IL-17 production was determined by flow cytometry, *Il9* and *Il23r* mRNA was determined **o**. *Bars* show + s.d. from combined six or more **a**, three **c**–**e**, **h**–**j**, two **k**, **l**, three **m**, **o** experiments. *P < 0.05, **P < 0.01, ***P < 0.001, ****P < 0.0001 (Student’s *t*-test and two-way analysis of variance)

We and others have shown that TGF-β1/IL-6-induced Th17 cells produce IL-9, which is suppressed by IL-23^5–7^. As compared to Th0, TGF-β1/IL-6-induced Th17 cells expressed higher levels of Foxo1 and IL-9 (Fig. 4a, b). While IL-23 enhanced IL-17, it suppressed *Il9* and *Foxo1* expressions in recall response and in vitro differentiated Th17 cells (Fig. 4c, d and Supplementary Fig. 5a). Consistently, reanalysis of published microarray data shows an increased *Il9* expression in *Il23r*−/− Th17 cells as compared to Wt Th17 cells (Supplementary Fig. 5b). We confirmed that *Il9* expression was enhanced in *Il23r*−/− as compared Wt mice upon immunization (Fig. 4e). Moreover, IL-23 exposure enhanced the phosphorylation of Foxo1 and AKT in Th17 cells (Fig. 4f and Supplementary Fig. 5c), which possibly suppressed IL-9 in Th17 cells.

Consistently, CA-AKT enhanced IL-17 while decreased IL-9 in Th17 cells (data not shown). We further validated the effect of PI(3)K/AKT-inhibition on the induction of IL-9 and IL-17 in Th17 cells. PI(3)K/AKT-inhibition enhanced IL-9 while suppressed IL-17 and Th17 cells-associated genes, *Il23r*, *Rorc*, *Gmcsf* and *Ahr* in Th17 cells (Fig. 4g–j and Supplementary Fig. 6a, b). Foxo1 inhibition reversed the effect of PI(3)K/AKT inhibition on enhancing IL-9 or suppressing IL-17 in Th17 cells (Fig. 4k, l and Supplementary Fig. 6c).

Other than AKT, SGK1 regulates Foxo1 functions in Th17 cells^18^. Reanalysis of published microarray data revealed *Il9* ranked second among the top 250 differentially expressed genes in *Sgk* ^−/−^ as compared to wt Th17 cells^18^. While Th17 cell-associated genes, *Il17a*, *Rora*, *Il1r* and *Ahr*, were downregulated, Th9-cell-associated genes, *Il9*, *Il2*, *Il21* and *Gata3*, were upregulated in *Sgk1* ^−/−^ Th17 cells (Supplementary Data 2 and Supplementary Fig. 6d), suggesting that upstream kinase of Foxo1 regulate IL-9 induction in Th17 cells. We further tested the effect of direct inhibition of Foxo1 in Th17 cells. Foxo1 inhibition either by Foxo1-shRNA or Foxo1 chemical inhibitor selectively inhibited *Il9* while enhanced *Il17* and Th17-associated genes in Th17 cells (Fig. 4m–o). Consistently, Foxo1 directly suppressed the RoRγt-mediated transactivation of *Il17a* and *Il23r* gene (Supplementary Fig. 7a, b), suggesting that Foxo1 inhibiting Th17 cell program by targeting Rorγt.

### Foxo1 controls IL-9 induction in TGF-β1-induced iTreg cells

**Fig. 5:**
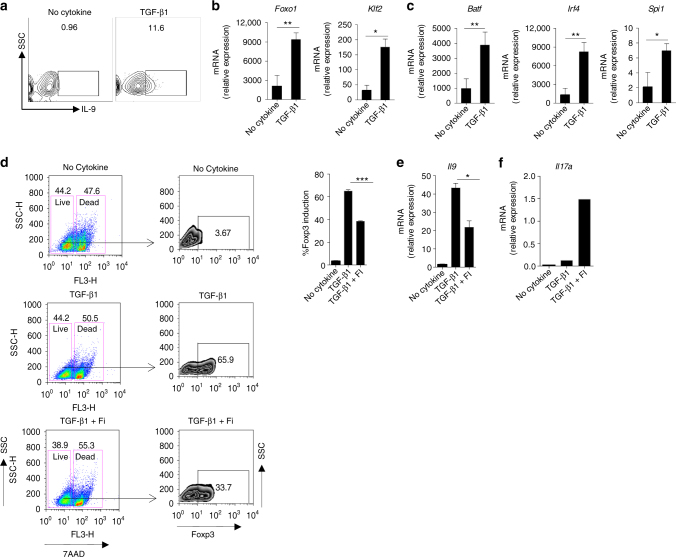
Foxo1 controls IL-9 induction in TGF-β1-induced iTregs. **a**–**c** Naive CD4^+^ T cells were activated with plate bound anti-CD3 and anti-CD28 in the presence or absence of TGF-β1. **a** 72 h later, cells were restimulated with PMA/Ionomycin for 5–6 h and then intracellular staining for IL-9 was performed, data representative of one of two independent experiments. At 72 h RNA was prepared from above cultured cells and qPCR was performed for *Foxo1* and *Klf2* **b**, *Batf*, *Irf4* and *Spi1* **c**. Naive T cells were activated with anti-CD3 and anti-CD28 in the presence of TGF-β1 with or without Foxo1 inhibitor (25 nm), *Foxp3* expression was determined by flow cytometry at 72 h, bars to the right shows % Foxp3 induction **d**, percentage CD4^+^Foxp3^+^ cell were determined by FACS. **e**, **f**, mRNA expression of *Il9* and *Il17* was determined by qPCR from above experiments. *Bars* show mean ± s.d. from combined three **b**–**e** experiments. *P < 0.05, **P < 0.01, ***P < 0.001 (Student’s *t*-test)

It has been shown that TGF-β1 stimulation of T cells not only induces Foxp3 expression but also induce IL-9^13^. Moreover, it is suggested that there are overlapping transcriptional similarities between Th9 and iTregs due to the presence of TGF-β1 in Th9 differentiating conditions^13^. Interestingly, Foxo1 was shown to be essential nTregs as well as iTregs functions^21–23^. Our data established a link between Foxo1 in IL-9 induction in Th9 and Th17 cells. Based on these, we wanted to test whether Foxo1 is required for IL-9 induction in iTregs cells. To test this, we differentiated sorted naive CD4^+^ T cells into iTregs in the presence of TGF-β1, and found that iTregs induce the expressions of IL-9, *Foxo1* and *Klf2*, a Foxo1 target gene (Fig. 5a, b). Moreover, iTregs also induced the expression of other Th9-associated transcription factors, *Batf*, *Irf4* and *Spi1* (Fig. 5c). We further tested the requirement of Foxo1 in IL-9 induction in iTregs, to do this, we differentiated iTregs in the presence and absence of Foxo1 inhibitor. It is known that deficiency of Foxo1 inhibits TGF-β1-mediated induction of Foxp3, which lead to enhance effector T cells functions. Consistently, our data indicated that Foxo1 inhibition suppressed the induction of Foxp3 significantly (Fig. 5d) without affecting cell survival as shown by live and dead population (Fig. 5d). We further tested IL-9 induction in iTregs in the presence Foxo1 inhibition, we found that Foxo1 inhibition significantly suppressed IL-9 in iTregs cells while enhanced IL-17 induction (Fig. 5e, f). In addition, we also found that Foxo1 inhibition also suppressed IL-9 in Th2 cells without affecting the IL-4 induction. Taken together, these data clearly indicated that Foxo1 is critically required for the IL-9 induction in iTregs.

### Foxo1 binds and transactivates IL-9 gene

**Fig. 6:**
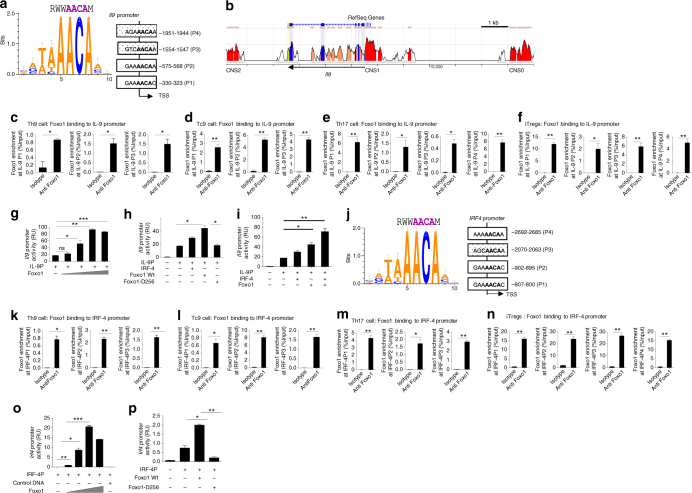
Foxo1 binds and transactivates IL-9 and IRF-4 promoters. **a** Bioinformatic analysis of Foxo1 motif in the proximal promoter of IL-9. **b** ECR browser-based analysis of IL-9 locus for Foxo1-binding sequence (5′-AACA-3′ core sequence) in conserved nucleotide regions between mouse and humans. **c**–**f** ChIP analysis of Foxo1 binding to IL-9 promoter in in vitro differentiated Th9 cells, Tc9 cells and Th17 and iTregs, results obtained with anti-Foxo1 antibody are presented as enrichment of Foxo1 at IL-9 promoter relative to input. **g**–**i** IL-9 promoter luciferase activity was determined in HEK293T cells transfected with IL-9P-driven luciferase reporter together with indicated plasmids, results were presented relative to the activity of a co-transfected control renilla luciferase reporter. **j** Bioinformatic analysis of Foxo1-binding site in IRF-4 promoter. **k**–**n** ChIP analysis of Foxo1 binding to the IRF-4 promoter in vitro differentiated Th9, Tc9, Th17 and iTregs. Results obtained with anti-Foxo1 antibody are presented as enrichment of Foxo1 at IRF-4 locus relative to input DNA. **o**, **p** IRF-4 promoter luciferase activity was determined in HEK 293T cells transfected with IRF-4-luciferase reporter plasmid along with Foxo1 plasmid (with increasing doses, **o**) and FoxoD256 **p** and firefly luciferase, results were presented relative to the activity of a co-transfected control renilla luciferase reporter (pRL-Tk). *Bars* show mean ± s.d. from combined three **c**–**f**, **k**, **l**, **n**–**p** and four **g**, **i** different experiments. *P < 0.05, **P < 0.01, ***P < 0.001 (two-tailed Student’s *t*-test)

Above data clearly suggested a critical association of Foxo1 and IL-9 in Th2, Th9, Th17 and iTregs. To further validate the association of Foxo1 with IL-9 mechanistically, we analyzed and identified four putative Foxo1-binding sites in proximal promoter of IL-9 (Fig. 6a)^33^. Among CNS1, CNS2 and CNS0 of IL-9 locus, we found the conserved consensus-binding sites of Foxo1 (5′-AACA-3′ core sequence) between mouse and human in CNS2 and CNS0 (Fig. 6b). Chip confirmed that Foxo1 binds to IL-9 promoter in Th9, Tc9 and Th17 cells and iTregs (Fig. 6c–f). Consistently, molecular signatures database (MSigDB) 3.0 suggested Foxo1 binding to IL-9, Stat5a/b, IRF1 and Samd3^34^, as Stat5, IRF1 and Smad3 are crucial for IL-9 induction in Th9 cells^3, 28, 35^. *Il9*-promoter-luciferase confirmed that Wt Foxo1, but not mutant Foxo1 (Foxo1D256; Foxo1 dominant-negative mutant), transactivates *Il9* promoter activity (Fig. 6g, h), which is synergistically enhanced by IRF-4, as IRF-4 known to transactivate *Il9* gene Fig. 6i).

It has been demonstrated that IRF-4 is required for the development of Th9 cells, and Foxo1-IRF4 interaction is essential in nutrient availability in adipocytes^36^. To understand the functional association of Foxo1 with IRF4 in IL-9 induction in Th9 cells, we found putative Foxo1 binding sites in proximal promoter of *Irf4* ^33^ (Fig. 6j). Chip analysis confirmed Foxo1 binding to *Irf4* promoter in Th9, Tc9, Th17 and iTregs (Fig. 6k–n). Wt Foxo1, but not mutant Foxo1, induced *Irf4* promoter activity possibly by their physical interactions at protein level (Fig. 6o, p and Supplementary Fig. 8a). Taken together these data indicated that in addition to IL-9, Foxo1 transactivates IRF-4 gene, which is crucial for IL-9 induction and Th9 development^11^.

### Foxo1 inhibition attenuates allergic inflammation in vivo

**Fig. 7:**
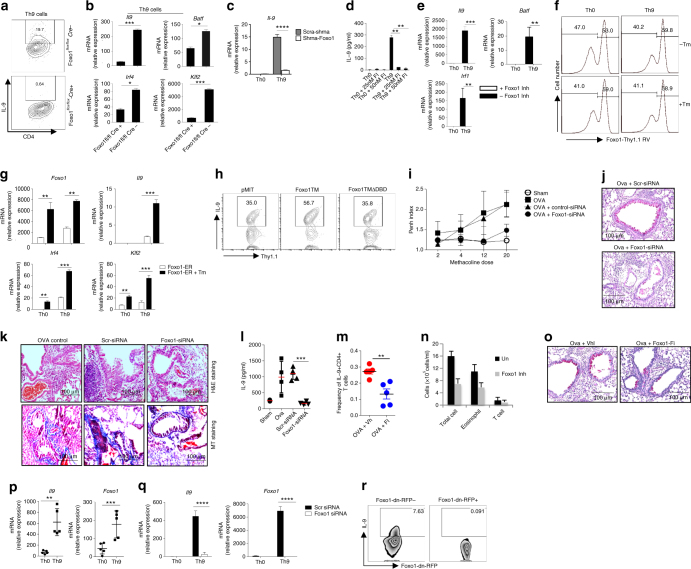
Foxo1 inhibition restricts allergic inflammation in asthma. Th9 cells were differentiated from Foxo1^*flox/flox*^ or Foxo1^*flox/flox*^-cre mice, IL-9 production was determined by intracellular cytokine staining **a**, mRNA expression for *Il9*, *Batf*, *Irf4* and *Klf2* was determined by qPCR **b**. Sorted naive CD4^+^ T cells were differentiated into Th9 conditions, and transduced with Scramble-shRNA or Foxo1-shRNA or treated Foxo1 inhibitor. *Il9* mRNA expression was determined with qPCR **c**, IL-9 production was quantified by ELISA **d**, mRNA expression of *Il9*, *Irf1* and *Batf* was determined by qPCR **e**. *Bars* show mean + s.d. from combined two **b**, **d**, three **c** and four **e** different experiments. *P < 0.05, **P < 0.01, ***P < 0.001, ****P < 0.0001 (Student’s *t*-test and two-way analysis of variance). s.d****. Naive T cells were transduced with Foxo-ER-Thy1.1, differentiated into Th9 condition, and treated with or without Tamoxifen. Thy1.1 surface expression was determined by FACS **f**, and mRNA expression of *Foxo1*, *Irf4*, *Il9* and *Klf2* was determined by qPCR on sorted Foxo-ER-Thy1.1^+^ or Foxo-ER-Thy1.1^−^ cells. *Bars* show mean + s.d. from combined two **g** experiments. **P < 0.01, ***P < 0.001 (two-way analysis of variance). **h** Sorted naive CD4^+^ T cells were transduced with pMIT-, Foxo1TM- or Foxo1TMΔDBD- Thy1.1^+^ retroviral vectors, IL-9 production was determined on Thy1.1^+^ cells by intracellular cytokine staining. Data representative is two independent experiments. **i** AHR activity was measured Penh index was calculated was determined in indicated group of mice with methacoline doses (*n* = 5). **j**, **k** H&E, MT and PAS staining in lung sections from above group of mice. **l** IL-9 ELISA in BAL fluid from mice treated with Foxo1- or Scr-siRNA (*n* = 5 mice per group), ***P < 0.001 (Student’s *t*-test). **m** Ova immunized Balb/c mice were treated with vehicle-control or Foxo1 inhibitor (20 mg kg^−1^), percentage of IL-9^+^CD4^+^ T cells was determined by intracellular cytokine staining in a recall assay (*n* = 5 mice/group). *Bars* show mean ± s.d. **P < 0.01 (Student *t*-test). **n** Total cells, eosinophils, T cells influx in BAL fluid were counted based on the total number of BAL cells. **o** PAS staining from the lung sections of Foxo1 inhibitor treated mice. **p** Sorted naive human CD4+ T cells were differentiated into Th9 cells. mRNA expression of *Il9* and *Foxo1* was determined by qPCR (*n* = 5 healthy individuals). *Bars* show mean ± s.d. **P < 0.01, ***P < 0.001 (Student’s *t*-test). Human Th9 differentiated cells transfected with Scr-siRNA or Foxo1-siRNA or dn-Foxo1-RFP, **q** mRNA expression of *Il9* and *Foxo1* was determined by qPCR at day 7 (*n* = 3 healthy individuals). *Bars* show mean ± s.d. ****P < 0.0001 (Student’s *t*-test). Intracellular IL-9 was determined in RFP^+^ and RFP^−^ cells, data representative of three healthy individuals **r**

To further validate the role of Foxo1 in IL-9 induction, we used genetic approach to delete Foxo1 in Th9 cells using Foxo1 conditional deficient system. Cre-mediated deletion of Foxo1 in Th9 cells substantially reduced IL-9, *Batf, Irf4* and *Klf2* (Fig. 7a, b). Similarly, Foxo1 shRNA and pharmacological inhibitor suppressed IL-9 and Th9-associated genes, *Irf1*, *Batf* and *Irf4* in Th9 cells (Fig. 7c, d). In addition shRNA-mediated inhibition of Foxo1 suppressed IL-9 induction in Tc9 cells (Supplementary Fig. 9a–e). We also found that inhibition of Foxo1 by siRNA suppressed IL-9 in Th2, Th9 and Th17 cells (Supplementary Fig. 10a). Moreover, Foxo1 inhibitor suppressed *Il9* expression without affecting *Il4* expression in Th2 cells (Supplementary Fig. 10b, c). Overexpression of Foxo1-enhanced *Il9*, *Klf2* and *Irf4* expression in Th9 cells (Fig. 7f, g). Consistently, constitutive active Foxo1 triple mutant (Foxo1TM, constitutive active (CA) Foxo1), which lack all three AKT-mediated phosphorylation sites, substantially enhance IL-9 while Foxo1TM-lacking DNA-binding domain (Foxo1TMDBD) failed to enhance IL-9 in Th9 cells (Fig. 7h). These observations suggest that DNA-binding activity of Foxo1 is essential for the induction of IL-9 in Th9 cells.

We tested in vivo functions of Foxo1 and its association with IL-9 in asthma since IL-9 and Foxo1 play crucial role in asthma^37, 38^. Foxo1 conditional deficient mice show an abnormal T cell activation, accumulate higher number of activated effector/memory T cells in secondary lymphoid organs. To avoid the interference of pre-activated T cells in asthma model, we used therapeutic approach to knock-down Foxo1 using siRNA in asthma model. While scr-siRNA treatment failed to attenuate AHR as compared to control group, Foxo1-siRNA treatment attenuated AHR significantly (Fig. 7i). Scr-siRNA-treated mice accumulated infiltrating inflammatory cells around the bronchi and vessel while Foxo1-siRNA treatment reduced accumulation of infiltrating cells (Fig. 7j). Histological examination confirmed that in vivo Foxo1-siRNA treatment attenuated bronchial hyperplasia of periodic acid-Schiff (PAS) positive goblet cells (important mediator of lung inflammation), when compared with Ova-challenge Scr-siRNA-treated mice (Fig. 7k). Foxo1-siRNA suppressed IL-9 production in BAL fluid as compared to Scr-siRNA group (Fig. 7l). Similarly, Foxo1-inhibitor attenuated Ova-induced asthma (unpublished observations), IL-9 induction and PAS-positive goblet cells (Fig. 7m–o). Foxo1 inhibitor reduced the total cell counts, eosinophil and T-cell number in asthma model (Fig. 7m). Finally, we confirmed that Foxo1 inhibition suppresses IL-9 induction in human Th9 cells (Fig. 7p, q). Inhibition of Foxo1 in human Th9 cells by Foxo1-dominant-negative-RFP inhibited IL-9 expression in Th9 cells (Fig. 7r).

Our study revealed an association of Foxo1 with IL-9-producing Th9 and Th17 cells, as Foxo1 binds and tranasctivates IL-9 gene. Loss or induction of Foxo1 by its direct inhibition or interference with its upstream kinases results in regulation of IL-9 induction in IL-9-producing T cells. Inhibition of Foxo1 not only suppressed IL-9 induction IL-9-producing T cells but also ameliorated development of asthma. Altogether these data thus identify Foxo1 as a crucial transcription factor for IL-9 induction not only in Th9 but also in other IL-9-producing T cells.

## Discussion

In summary, our global gene expression profiling identified the transcription factor Foxo1 differentially expressed and critically required for the induction of interleukin (IL) 9 in helper T (Th) 2, Th9, Th17 and iTreg cells. Our data suggests that the expression of Foxo1 reinforced the development and effector functions of Th9 cells in allergic inflammation. We further identified for the first time that Foxo1 promotes IL-9 while suppresses IL-17 and Th17-associated genes in Th17 cells. Our data identified upstream kinases, AKT that regulates IL-9 induction in Th9 and Th17 cells via Foxo1. Inhibition of AKT promotes IL-9 while suppresses IL-17 in Th17 cells via Foxo1. Mechanistically, Foxo1 binds to IL-9 and IRF4 promoter and transactivate their expressions in Th9 cells. Altogether, our data identified new pathway that is essential for the induction of IL-9 in Th9 and Th17, and thus could potentially be used in designing targeted therapies aimed at alleviating the course of IL-9-mediated allergic inflammation and cancer immunotherapy.

Once classified into Th1 and Th2 effector subsets, the subsets of effector T cells have been expanded to include most recently identified Th9 and Th17 cells. We and others have identified Th9 cells as distinct effector T cell subset arising from a cytokine combination of TGF-β1 and IL-4. Owing to the plasticity and their instability, effector Th subsets can inter-convert from one subtype to another^39^. For example, Th1 cells are closely related to Th17 cells as both of them express T-bet, a master transcription factor of Th1 lineage^17, 40^. In fact, Th17 cells are plastic and tend to convert into Th1 cells in vivo in T-bet and Stat4 dependent manner^41^. Similarly, Th9 subset is related to Th2 cells, as both of these subsets require IL-4 and share their downstream transcription factors such as STAT6 and GATA3 for their development^42, 43^. Although, Th1, Th2 and Th17 cells distinctly express lineage-specific transcription factor that promote their lineage-specific genetic program, the lineage-specific transcription factor for Th9 cells has not been identified. Nonetheless Th9 cells express IRF-4, PU.1, BATF and IRF-1 to induce their development, but none of these factors determines the lineage-specificity of Th9 cells developmental program. In fact IRF-4, PU.1, BATF and IRF-1 are co-expressed or shared by Th2 cells (IRF-4 and PU.1)^44, 45^, Th17 cells (IRF-4 and BATF)^14, 15^ and Th1 cells (IRF-1)^16^. In this report, we have identified Foxo1 as a transcription factor that is essential for IL-9 induction in Th9 cells and Th17 cells. Foxo1 negatively regulates the development of Th17 cells by directly repressing Rorc and IL-23R expression. Similarly, Foxo1 also inhibits the expression of IFN-γ in Treg cells^23^. Here we have demonstrated that Foxo1 mediate the expression of IL-9 in Th9 and Th17 cells while suppressing the expression IL-17 in and Th17 cells, respectively.

Th17 cells produce IL-9 albeit at lower levels as compared to Th9 cells, and moreover IL-9 promotes the development of Th17 cells^6^. Recent literature has suggested that IL-9 is expressed by non-pathogenic Th17 cells. IL-23-IL-23R signaling enhances pathogenicity of Th17 and suppresses IL-9 in pathogenic Th17 cells^17^. Consistently, our data indicated that TGF-β1/IL-6-induced Th17 cells expressed both IL-9 and Foxo1. Interestingly, exposure of IL-23 to TGF-β1/IL-6-induced Th17 cells suppressed the expression of IL-9 and Foxo1 and promoted Th17 cell phenotypes.

The mechanism by which IL-23 promotes the phenotypes of pathogenic Th17 cells has been elucidated elsewhere^17, 18^. It is suggested that IL-23 induces SGK1-mediated inactivation of Foxo1 to enhance the induction and effector functions of pathogenic Th17 cells^18^. Moreover, Foxo1 was shown to directly bind to Rorγt and repress IL-17 and IL-23R expression in Th17 cells^19^. Consistently, our data indicated that the inhibition of Foxo1 enhanced the expression of *Il17a*, *Rorc*, *Gmcsf* and *Il23r* while suppressing the expression of IL-9 in Th17 cells.

Our data also indicated that Th2 and iTreg cells expressed Foxo1 together with IL-9, and the expression of IL-9 was inhibited upon Foxo1 inhibition.

Phosphorylation of Foxo1 tightly controls its transcriptional activity by regulating its shuttling between nucleus and cytoplasm within the cell^46^. Upon phosphorylation by its upstream kinase, AKT, Foxo1 loses its transcriptional functions due to its nuclear exclusion^46^. Our data demonstrated that the inhibition of upstream PI(3)K/AKT pathway enhanced the development of Th9 cells with increased expression of Foxo1. Moreover, Foxo1 inhibition blocked the effect of PI(3)K/AKT inhibition on Th9 cells, suggesting the involvement of PI(3)K/AKT-Foxo1 axis in inducing the development of Th9 cells. We have further shown that the inhibition of PI(3)K/AKT axis enhanced IL-9 in Th17 cells while suppressed the development of Th17 cells by enhancing the transcriptional activity of Foxo1, which is in accordance to recent findings that the inhibition of PI(3)K/AKT pathway suppressed the differentiation of Th17 cells^19^. Strikingly, our data demonstrated that the inhibition of PI(3)K/AKT axis reinforced the Th17 cells to produce IL-9 in Foxo1-dependent manner. We further demonstrated that the inhibition of Foxo1 not only suppressed IL-9 production but also rescued IL-17 production suppressed by PI(3)K/AKT inhibition.

PU.1 was shown to be required for the induction of IL-9 in Th2 and Th9 cells. In fact the ectopic expression of PU.1 was shown to convert Th2 cells into IL-9 producers^12^. However, the PU.1 had modest effect on IL-9 induction in iTregs and Th17 cells. Nonetheless the PU.1 can bind *Il9* promoter directly in Th9 cells^12^. Unlike PU.1, our data indicated that Foxo1 binding to IL-9 promoter in IL-9-producing Th9, Th17 and iTregs. Our data further demonstrated that inhibition of Foxo1 suppressed IL-9 induction in Th2, Th9 and Th17 and iTregs. Although the role PU.1 and Foxo1 axis is not clearly understood in Th cells differentiation, the functions of Foxo1 and PU.1 were clearly demonstrated in the biology of B cell development. In fact, Foxo1 were shown to be upstream to PU.1 in pro B cell stage, as Foxo1 deficiency reduces the expression of PU.1^47^. Consistently, our data clearly indicated the upstream role of Foxo1 as compared to PU.1 in IL-9 induction in Th9 cells, as inhibition of Foxo1 reduced the mRNA expression of PU.1. Since PU.1 alone is not sufficient to drive IL-9 induction in iTregs and Th17 cells, it will be interesting to determine whether PU.1 and Foxo1 can together synergistically promote IL-9 in Th9, iTregs and Th17 cells.

Our data indicate that Foxo1 can directly bind to *Il9* promoter in Th9, Tc9 and Th17 cells, and ectopic expression of Foxo1-enhanced Th9 cells characteristics by enhancing IL-9 and IRF4 expression. Since IRF-4 has been shown to be essential for IL-9 induction and Th9 development^11^, therefore it might be possible that Foxo1 binding and transactivating IRF4 gene locus in Th9 cells is prerequisite for Th9 cells. Our data suggested that Foxo1 binds and transactivates IRF-4 promoter, and ectopic expression of Foxo1 increases IRF-4 expression in Th9 cells. This data is in accordance with the finding where Foxo1 directly induces IRF4, and together these factors create insulin-repressible feed-forward loop in adipocytes^36^. Our data, also suggested that Foxo1 and IRF4 synergistically enhance the transctivation of IL-9 possibly due to IRF-4-Foxo1 interaction at protein level.

IL-9 is known to play crucial role in allergic inflammation in asthma as both IL-9 and IL-9R were shown to be genetically associated with the disease^48–51^. Furthermore, transgenic overexpression of IL-9 in lung induced severe allergic inflammation and asthma^52^. Intranasal administration of anti-IL-9 antibody suppresses the severity of murine asthma. Consistently, our data suggested that the blocking of Foxo1 strongly suppressed the signs of allergic inflammation in mice model. Our data further supported that in vivo blocking of Foxo1 reduces IL-9 production in lung-infiltrating CD4^+^ T cells. In addition, Foxo1-deficient T cells failed to produce IL-9. however we did not use Foxo1-CD4 conditional deficient mice in asthma model, as these mice harbor multiple defects including activated T cells phenotype at early age with defects in Foxp3^+^ Tregs functions^22, 23, 53^. In addition, Foxo1-CD4-conditional deficient mice also accumulate significantly higher frequency of Th17 cells and leads to multiple organ failure^19, 21, 22^. Foxo1 is crucial for T-cell homeostasis and trafficking, as its deficiency down regulates CCR7 and KLF2. KLF2 is the transcription factor that is required for the expression of T cells homing markers, CD62L and S1PR1. In addition, Foxo1 controls expressions of cyclin-dependent kinase inhibitor, P27KIP1, and IL-7Ra^25, 54, 55^. Since Foxo1 intrinsically controls the expression of the molecules crucial for cell homing, cell cycle and homeostatic T-cell survival and proliferation of naive T cells, therefore transferring Foxo1KO T cells in Rag would still lead to the similar activated/aberrant phenotypes as Foxo1CKO mice.

Because of these defects and activated phenotypes of T cells in Foxo1-CD4-conditional deficient mice, we have used alternate approach to therapeutically block Foxo1 using Foxo1-siRNA or Foxo1-chemical inhibitor in experimental allergic inflammation model of asthma. Our data demonstrated therapeutic efficacy of Foxo1-siRNA and Foxo1 inhibitor in inhibiting IL-9 and ameliorating signs of ova-induced asthma in mice. In fact, our data are consistent of recently published study showing that Foxo1-chemical inhibitor suppressed airway inflammation^38^. Similar to mice, our data demonstrated that Foxo1 is required for the induction of IL-9 in human Th9 cells, as inhibition of Foxo1 in human Th9 cells also suppressed IL-9.

In summary, we have shown for the first time that Foxo1 acts as a key transcription factor in the biology of Th9 and IL-9-producing T cells. Expression of Foxo1 is required for the induction of IL-9 while suppresses IL-17 in Th17 cells. Moreover, the inhibition of AKT enhances the development of Th9 cells mediated via Foxo1. Given the proinflammatory functions of Th9 cells in allergic inflammation, autoimmunity and tumor immunity, the identification of Foxo1 as a key transcription factor that dictates the development and effector functions of Th9 and IL-9-producing T cells could prove beneficial in designing targeted therapies aimed at alleviating the course of autoimmune diseases and anti-cancer therapy.

## Methods

### Mice

C57BL/6 (#000664), Balb/c (#000561) wild-type (Wt) and *Nos2*−/− (#002596) mice were procured from the Jackson Laboratory, housed and maintained in a conventional pathogen- free small-animal facility at Translational Health Science and Technology Institute, National Institute of Immunology (NII), New Delhi, India and Institute of Genomics and Integrative Biology (IGIB), New Delhi, India. All the mice used in the experiments were 8–12 weeks and were age- and sex-matched. All experiments were performed in accordance to the approved guidelines outlined by Institutional Animal Ethics Committee of the National Institute of Immunology, Institute of Genomics and Integrative Biology (IGIB) and Translational Health Science and Technology Institute (THSTI). Experiments on Foxo1-conditional^23^ and *Il23r*−/−^56^ mice were performed at Harvard Medical School in accordance to the guidelines outlined by the Harvard Medical Area Standing Committee on Animals at Harvard Medical School. All human experiments were performed in accordance to the approved guidelines of Human Ethics Committee of THSTI, Faridabad and AIIMS, New Delhi. Human blood samples were collected from healthy individuals after the written informed consent. Briefly, healthy volunteers were enrolled in this study based on the inclusion and exclusion criteria prescribed by the Human Ethics Committee.

### Plasmids and constructs and antibodies

Following Foxo1 constructs were procured from Addgene and used in the study - pCMV5 HA-Foxo1 (kind gift of Domenico Accili, Addgene, plasmid 12142), Myc Foxo1 D256 (kind gift of Domenico Accili, Addgene 12145), FLAG-Foxo1 ADA (kind gift of Domenico Accili, Addgene 12149), MSCV-IRES-GFP (kind gift of Tannishtha Reya, Addgene 20672), pMIG (kind gift of William Hahn, Addgene 9044), MSCV-IRF4 (Kind gift of Vijay K. Kuchroo, Harvard Medical School), Foxo-ER-Thy1.1 (Kind gift of Mark S Schlissel), pCMV5 HA Akt DN (Addgene 16243; Mien-Chie Hung), IL-17 2Kb and IL-17CNS.5 (kind gift of Warren Strober, Addgene, plasmid 20124), IL-23R-Luciferase (kind gift of Kojiro Sato), IL-9-Lucifease (kind gift of Edgar Schmitt), IRF-4-luciferase (kind gift of Evan D. Rosen), pBABE RFP-DN-Foxo1 (Kind gift of Kevin Janes, Addgene 45813). Full-length mouse AKT- dominant-negative (DN) gene was excised from pCMV-Akt-DN (kind gift of Addgene; Plasmind16243) using restriction enzyme *Bgl*II and *EcoR*I and cloned into pMIG Vector (kind gift of William Hahn, Addgene 9044). Mouse IRF-4 was cloned into pCMV-6 Entry Vector (for DDK-IRF-4) between restriction sites *Hind*III and *Xho*I by using the primers- 5′-GCATAAGCTTATGAACTTGGACGGGC-3′ and 5′-GCATCTCGAGCTCTTGGATGGAAGAATGACG-3′. pMIT, Foxo1TM/pMIT and DNA-binding deficient mutant-Foxo1 TMΔDBD/pMIT were a kind gift of Dr Celine Charvet.

Mycoplasma-free HEK293T (ATCC CRL-3216) cell lines used in this study were procured from ATCC. Cell lines were routinely tested for mycoplasma contamination in the laboratory. None of the cell lines used in the study were listed or misidentified in the database of ICLAC or NCBI Biosample.

### GEO2R and microarray reanalysis

Microarray publicly available via Gene Expression Omnibus (GEO) were reanalyzed using the built-in GEO2R software. The differential gene expression between Th2 vs. Th9 cells (GEO accession code, GSE44937; Supplementary Data 1)^13^, Wt and *Sgk−*/*−* Th17 cells GEO accession code, GSE43956; Supplementary Data 2)^18^ and pathogenic (TGF-β3/IL-6, IL-1β/IL-6/IL-23) vs. non-pathogenic (TGF-β1/IL-6) Th17 cells (GEO accession code, GSE39820) was determined by GEO2R. My Pattern Finder (RegAnalyst) was used to identify Foxo1 binding sites in IL-9 and IRF-4 promoters^33^.

### In vitro mouse T-cell differentiation

Single cell suspensions were made from from spleen and lymph nodes of 6–8 weeks old mice. CD4^+^ and CD8^+^ T cells were purified using CD4 (L3T4) MicroBeads, mouse (#130-049-201, Miltenyi Biotech) and CD8a (Ly-2) MicroBeads mouse, (#130-049-401, Miltenyi Biotech). CD4^+^ and CD8^+^ T cells were further sorted using f﻿luorescence-activated cell sorting (FACS) on BD FACSAriaIII (BD Biosciences) to obtain naive CD4^+^CD62L^+^CD44^−^T cells and CD8^+^CD62L^+^CD25^−^T cells using anti-CD4-Percp (RM4-5, BioLegend), anti-CD62L-APC (MEL-14, BioLegend), anti-CD44-PE/Cy7 (IM7, BioLegend) and anti-CD8-FITC (53–6.7, BioLegend). The purity of sorted cells was typically ~98% in post-sort analysis. Sorted cells were activated with plate-bound anti-CD3 (2.0 µg ml^−1^, 145-2C11, BioXcell) and anti-CD28 (2.0 µg ml^−1^, PV1, BioXcell). The cells were differentiated into Th2 conditions by adding rmIL-4 (10 ng ml^−1^) plus anti-IFN-γ (10 μg ml^−1^, XMG1.2, BioXcell), or Th9 conditions by adding rhTGF-β1 (2.0 ng ml^−1^) plus IL-4 (20 ng ml^−1^) or Th17 conditions by adding rhTGF-β1 (2.0 ng ml^−1^) plus rmIL-6 (25 ng ml^−1^). Cells were usually harvested on day 3 for RNA, intracellular cytokine staining, and flow cytometry analysis for intracellular cytokine staining. Wherever mentioned, Foxo1 inhibitor (AS1842856) (Calbiochem; 25 nM), PI(3)K/AKT inhibitor (LY294001) (Calbiochem; 5.0–10 μM), were added at start of the culture. Total CD4^+^ T lymphocytes were isolated from ova-induced asthma mice and were in vitro re-primed with ova in presence or absence of LY294002 under Th9 culture conditions.

### In vitro human T cell differentiation and nucleofection

Human Th9 cells were generated as described^5^. Briefly, human PBMCs from healthy donors were isolated by Ficoll-paque (GE Healthcare) gradient, and labeled with anti-CD4-APC (RPA-T4, BioLegend), anti-CD45RA-PE/Cy7 (HI100, BioLegend) and anti-CD45RO^−^ Percp Cy5.5 (UCHL1, BioLegend) in 1: 500 dilution, and then naive human CD4^+^ T cells (CD4^+^ T CD45 RA^+^ CD45RO^−^ were sorted using BD FACSAriaIIII (BD Bioscience). The purity of sorted cells was typically ~98% in post-sort analysis. Sorted naive T cells were activated with plate bound anti-CD3 (10 μg ml^−1^, OKT-3, BioXcell) and soluble anti-CD28 (3.0 μg ml^−1^, #555725; BD Bioscience) in round-bottom 96 well plate. For differentiation of Th9, TGF-β1 (2.0 ng ml^−1^, #100-21 C, PeproTech), IL-4 (20 ng ml^−1^, #200-04, PeproTech), IL-2 (50 U ml^−1^, #200-02, PeproTech) were added to the cell culture. For the induction of Th17 cells differentiation, TGF-β1 (2.0 ng ml^−1^, #100-21 C, PeproTech), IL-6 (25 ng ml^−1^, #200-06, PeproTech), IL-21 (20 ng ml^−1^, #200-21, PeproTech), IL-23 (20 ng ml^−1^, #200-23, PeproTech) and IL-1β (10 ng ml^−1^, #200-1B, PeproTech) were used. Cells were cultured for 6 days and analyzed by intracellular cytokine staining. Sorted naive human CD4^+^T cells were nucleofected in 100ul of Amexa T cell solution (#V4XP-3024, Lonza) with pBABE-RFP-dn-Foxo1, these transfected T cells were further differentiated into Th9 cells and analyzed at day five for RFP and IL-9 expression.

### Cytokine analysis and real-time PCR

Culture supernatants were collected on day 2 and cytokines were measured by enzyme-linked immunosorbent assay (ELISA) as described^17^. On day 4 (unless noted otherwise) after culture, RNA was extracted with an RNeasy kit (#74104, Qiagen; # Mdi), then was reverse-transcribed with an iScript cDNA Synthesis kit (#1708891, Bio-Rad) and analyzed by quantitative PCR with a Fast 7500 Dx Real-time PCR system (Applied Biosystems) with the following primers and probes (from Applied Biosystems; identifier in parentheses): *Il17a* (Mm00439618_m1), *Il17f* (Mm00521423_m1), *Ifnγ* (Mm01168134_m1), *Il9* (Mm00434305_m1), *Il4* (Mm03682085_m1), *Csf2* (Mm01290062_m1), *Tbx21* (Mm00450960_m1), *Ahr* (Mm00478932_m1), *Il22* (Mm00444241_m1), *Il23r* (Mm00519943_m1), *Rorc* (Mm00441144_g1), *Rora* (Mm00443103_m1), *Foxo1* (Mm00490672_m1), *Irf4* (Mm00516431_m1) and *G* *apdh* (Mm99999915_g1), *Batf, Irf1* (Mm01288580_m1), *Spi1, Klf2, Foxo3a* The comparative threshold cycle method and an internal control (*Gapdh*) were used for normalization of the target genes.

Results were analyzed with SDS 2.1 software. The cycling threshold value of the endogenous control gene (Gapdh) was subtracted from the cycling threshold value of each target gene to generate the change in cycling threshold (ΔCT). The relative expression of each target gene is expressed as the ‘fold change’ relative to that of un-stimulated samples (2 − ΔCT). We used the previously used formula (POWER(2,−ΔCT) ^∗^ 10,000)^17^ to calculate the relative expression of gene expression in this manuscript.

### Intracellular staining of cytokines and phospho proteins

Cells were stimulated for 4 h with PMA (phorbol 12-myristate13-aceate; 50 ng ml^−1^; Sigma-Aldrich) and ionomycin (1.0 µg ml^−1^; Sigma-Aldrich) and a protein-transport inhibitor containing monensin (#554724 GolgiStop, BD Biosciences) before detection by staining with antibodies. Surface markers were stained for 15–20 min in room temperature in PBS with 1% FCS, then were fixed in Cytofix and permeabilized with Perm/Wash Buffer using Fixation Permeabilization solution kit (#554714, BD Biosciences) and stained anti-IL-17A (TC11-18H10, BioLegend); anti-IL-9 (RM9A4, BioLegend), anti-IFN-γ (XMG1.2, BioLegend) diluted in Perm/Wash buffer as described^17^.

For analysis of signaling via phosphorylated AKT, Foxo1 proteins by flow cytometry, Cells were stimulated as mentioned, cells were fixed with Cytoperm/Cytofix (BD Biosciences) and permeabilized with Perm/Wash Buffer (BD Biosciences), then stained with antibody to phosphorylated AKT, Foxo1 and Total Foxo1, antibody or isotype-matched control antibody (#DA1E, Cell Signaling Technology). For Foxp3 staining, cells were fixed with a Foxp3/ Transcription factor Fixation/Permeabilization Concentrate and Diluent (eBioscience) and then stained with anti-Foxp3 antibody (FJK-16, eBioscience). All antibodies were used in a 1:500 dilution. The cells were by flow cytometry using a FACSVerse (BD Biosciences), data were analyzed with FlowJo software (Treestar)^17^.

### Retroviral transduction

Retroviral transduction of T cells was performed as described earlier^17^. For preparation of pseudo-typed viruses, HEK293T were cultured at a density of 1 × 10^6^ in 10 cm dishes. Next day, cells were transfected with 10 ug of GAG-POL, 10 μg of PCL and 10 μg of respective retroviral constructs- Foxo-ER-Thy1.1, pMIG-Akt-DN, MSCV-CA-AKT, pMIT, Foxo1TM/pMIT, Foxo1 TMΔDBD/pMIT. Viral supernatants were harvested after 72 h of transfection. Retroviral expression constructs were transfected into human embryonic kidney (HEK) 293T cells along with eco and gag-pol viral envelope constructs^17^. Viral supernatants were collected between 60–72 h after transfection, and were added to primary T cells that had been activated for 12–16 h with plate-bound anti-CD3 (2.0 µg ml^−1^; 1452C11; Bio Xcell), anti-CD28 (2 µg ml^−1^; PV1; BioXcell) and cytokines for Th9 conditions as mentioned above. Cells were spun for 60 min at 32 °C at 2000 r.p.m. in the presence of polybrene (8.0 µg ml^−1^; Sigma) and were incubated for 3 days at 37 °C. Foxo1-ER Thy1.1^+^ or AKT-DN-GFP+ cells (expressing empty retroviral vector or retroviral vector encoding pMSCV-IRES-GFP, or pMSCV-IRES-Thy-1.1) were detected 2–3 days after infection. T cells infected with Foxo-ER-Thy1.1 were treated with or without Tamoxifen (20 nM; Sigma). Sorted Foxo1-Thy-1.1^+^ or Thy-1.1^+^ cells were processed for qPCR analysis for indicated genes.

### siRNA transfection in primary T cells

Naive CD4^+^ T cells were transfected with silencer select predesigned 50 nM siRNA specific for mouse *Foxo1* (#AM16810, Ambion, Life Technologies) or silencer negative control siRNA (#AM4611, Ambion, Life Technologies) with transfection reagent (#MIR 2155, Trans-IT-TKO Transfection Reagent, Mirus)^3^ according to the manufacturer’s instruction. 24 h after transfection, cells were stimulated with anti-CD3 and anti-CD28 and differentiated towards Th0, Th9 and Th17 conditions in presence or absence of LY294002 as indicated. Human Th9 cells were transfected with human Foxo1-specific siRNA or scramble siRNA and were stimulated for 4–5 days before analysis of IL-9 by intracellular cytokine staining.

### Luciferase reporter assay

DNA X-Treme Gene 9 (#6365779001, Sigma Aldrich) was used to transiently transfect HEK293T cells (4 × 10^4^; 48 well) with specified expression vectors, empty vector controls, promoter firefly luciferase reporter vector and renilla luciferase reporter vector Luminiscence for luciferase expression was measured after 48 h of transfection by luciferase reporter assay system (#E2940, Dual-Glo Luciferase Assay System, Promega). Briefly, 293T cells were transfected with 150 ng of the reporter vector as indicated (*Il23r*, *Il17a*, *Il17CNS.5*, *Il9* and *Irf4*) coding for firefly luciferase (pGL3basic; Promega) under the control of *Il9* promoter and with 150 ng of expression vectors as mentioned in the manuscript. Cells were cultured for 48 h before lysing. Firefly luciferase activity was normalized with renilla luciferase activity and the result was represented as RLU. Following constructs were used in the luciferase assay: *Il9* promoter luciferase, HA FOXO, pCDNA EGFP, pCDNA IRF4, FOXO ADA and MF D256 (Domenico Accili; Addgene 12145).

### ChIP PCR

Sorted naive CD4^+^ and CD8^+^ T cells were polarized into Th9 or Tc9 cells with TGF-β1 plus IL-4 for 72 h. Cells were cross-linked, fixed and processed with Simple ChIP Enzymatic Chromatin IP Kit (Magnetic Beads) (#9003 S, Cell Signaling Technology) according to the manufacturer’s instructions. Cell lysates were Immuno-precipitated with anti-Foxo1 antibody (#39670, Abcam) and rabbit IgG ChIP grade (#ab46540, Abcam). Region of *Il9* and *Irf4* promoter containing putative Foxo1-binding sites were amplified by SYBR Green chemistry (#KK4615, Kapa SYBR Fast). Results were quantified relative to percent input. List of the primers used for amplification are mentioned in Supplementary Table 1. Position of Foxo binding motif with respective to TSS (transcription start site) are depicted for *Il9* and *Irf4* promoters in Fig. 6a, j, respectively.

### Transcriptome profiling using RNA quantification sequencing

To understand the molecular mechanism of Th0 and Th9, RNA derived from both cells were subjected to next-generation sequencing (NGS) to generate deep coverage RNASeq data. Sequencing libraries of Poly A selected mRNA were generated from the double-stranded cDNA using the Illumina TruSeq kit according to the manufacturer’s protocol. Library quality control was checked using the Agilent DNA High Sensitivity Chip and qRT-PCR. High-quality libraries were sequenced on an Illumina HiSeq 2500. To achieve comprehensive coverage for each sample, we generated ~25–30 million paired end reads.

### RNASEQ data analysis

Sequencing data were processed to remove any adaptor, PCR primers and low-quality transcripts using FASTQC and fastx. These high-quality, clean reads were aligned against human genome using tophat2 and bowtie2 packages (http://tophat.cbcb.umd.edu/). Gene expression measurement was performed from aligned reads by counting the unique reads using htseq-count algorithm. The read count based gene expression data was normalized on the basis of library complexity and gene variation using the R package EdgeR. The normalized count data was compared among groups using a negative binomial model to identify differentially expressed genes. The differentially expressed genes were identified on the basis of multiple-test corrected *P* value and fold change. Genes were considered significantly differentially expressed if the *P* value was <0.0001 FDR and absolute fold change >2^57, 58^. Unsupervised analysis was performed using Principal Component Analysis (PCA) and hierarchical clustering on preprocessed normalized data^59^. PCA projects multivariate data objects onto a lower dimensional space while retaining as much of the original variance as possible.

### Regulatory module analysis

The regulatory module analysis was used to identify the cascade of upstream transcriptional regulators that can explain the observed gene expression changes in Th9 to identify key regulators (master regulators) and understanding underlying biological mechanism^60^. The analysis will help in identifying first which transcription regulators are significantly affected by the Th9 vs Th0 comparison as well as determining whether they are activated or inhibited. The activation or inhibition of transcriptional regulators was determined by determining the overlap among users data with activation or inhibition signatures of regulators. The significance of overlap was determined using one tailed fisher Exact test.

### Induction of allergic airway inflammation in mice

The mice model of experimental AAI was induced by administering three intra-peritoneal (i.p.) injections of 50 µg ovalbumin (OVA) that were adsorbed in 2 mg alum on days 0, 7 and 14 followed by whole body exposure to ovalbumin aerosol (3%) from days 21 to 27. On days 24 and 26, 50 mg kg^−1^ of Scr-siRNA or Foxo1-siRNA was administered through intranasal (with isoflurane anesthesia). On day 28, AHR was estimated using Buxco plethysmography using different concentrations of methacholine as described earlier^61^. Briefly, mice were acclimatized in a single chamber plethysmograph for 5–10 min. After the acclimatization, enhanced pause (Penh) readings were determined in these mice for 2 ½ min and then they were exposed to aerosols of either PBS or increasing concentrations (75 µl volume, 30% duty cycle) of methacholine (Mch) to get baseline and Mch-induced Penh, respectively. Penh, an unit less parameter, has been demonstrated to be valid in Balb/c mice in which AHR is mostly due to peri-bronchial inflammation. Individual variations in mice were normalized by converting baseline Penh to 1. After the measurement of AHR, mice were euthanized and bronchoalveolar lavage was performed to get both BAL fluid supernatants and cell pellets. The resultant pellets were used for estimating differential and total cell count^62^. Then the lungs, lymph nodes and spleens were harvested. One portion of each mouse lung was fixed, paraffin embedded, and sectioned. These sections were stained with various staining like H&E (airway inflammation), periodic acid-Schiff (goblet cell metaplasia) and masson’s trichrome staining (sub-epithelial fibrosis). Inflammation scoring was performed with experimentally blind investigators as described^62^ and the results were shown as perivascular (PV), peribronchial (PB) inflammation and total inflammation. BAL cells were stained with leishman’s staining and results were shown as absolute cells by multiplying the total cell count and absolute cell count.

### Statistics GraphPad

Prism 5.0 was used for statistical analysis (linear regression with 95% confidence interval, and unpaired, two-tailed Student’s *t*-test and analysis of variance). We have used unpaired two-tailed Student’s *t*-test for all comparisons however for the data with multiple comparisons and grouped analysis, we have used analysis of variance. We have used one tailed fisher Exact test to calculate the significance of overlap in transcriptional regulators in RNAseq data. Differences were considered statistically significant with a *P* value of less than 0.05.

### Data availability

Sequence data that support the findings of this study have been deposited in GEO with the primary accession code GSE100634. Publically available data with accession code, GSE44937, GSE43956, were reanalyzed with GEO2R software. The authors declare that all other data supporting the findings of this study are available within the article and its supplementary information files.

## Electronic supplementary material

Supplementary information

Supplementary data 1

Supplementary data 2
