# Supplementary material for: Transcription factor Foxo1 is essential for IL-9 induction in T helper cells
Source: Nat Commun. 2017 Oct 9;8:815. doi: 10.1038/s41467-017-00674-6 (PMC5634439; doi:10.1038/s41467-017-00674-6)
Supplement: Supplementary file 3 — Supplementary data 2 [file 41467_2017_674_MOESM3_ESM.pdf]

Description of Supplementary Files

File Name: Supplementary Information

Description: Supplementary figures, supplementary table

File Name: Supplementary Data 1

Description: Top 250 differentially expressed genes in Th9 cells as compared to Th2 cells (GEO GSE44937)

File Name: Supplementary Data 2

Description: Top 250 differentially expressed genes in Wt and Sgk-/- Th17 cells of reanalyzed microarray data (GSE43956) using GEO2R

Supplementary Figure 1

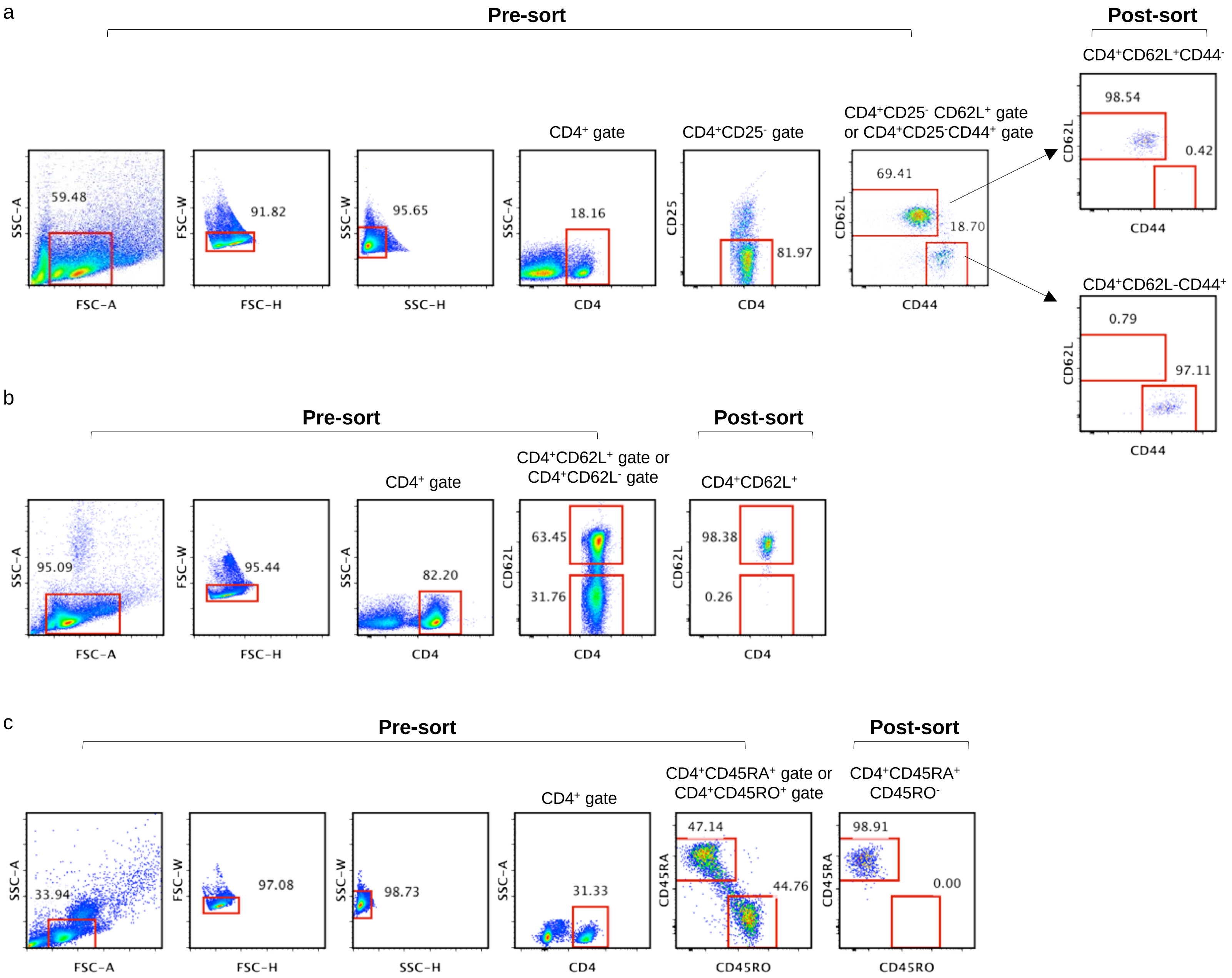

Supplementary Figure 1

d gating strategy for fig. 2 g

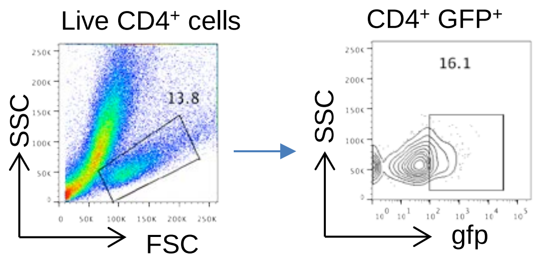

e gating strategy for fig. 2 h

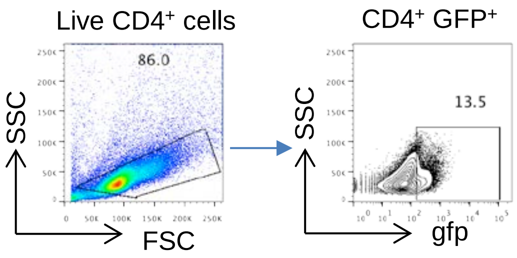

f gating strategy for fig. 3 e

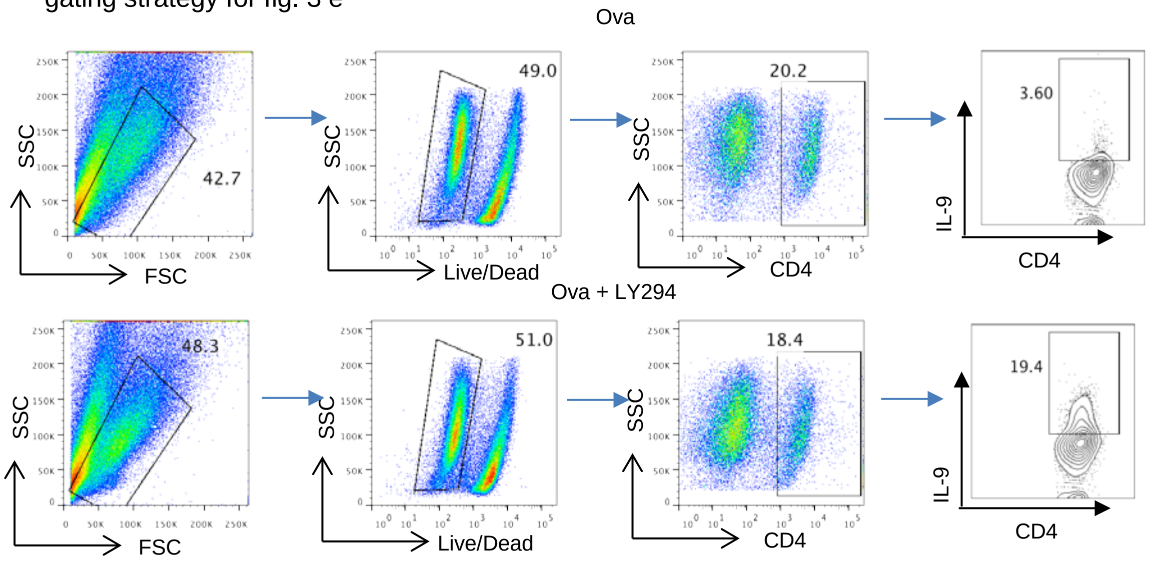

g

gating strategy for fig. 4 n

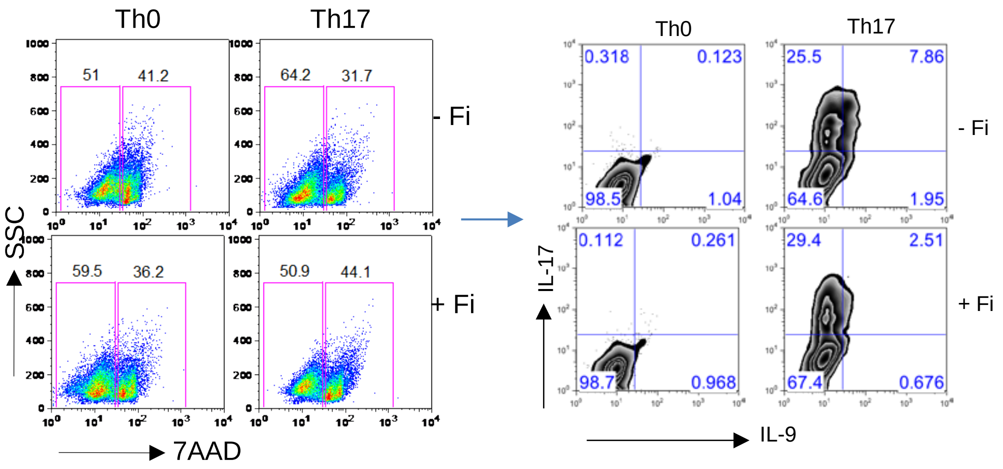

h

gating strategy for fig. 6 f

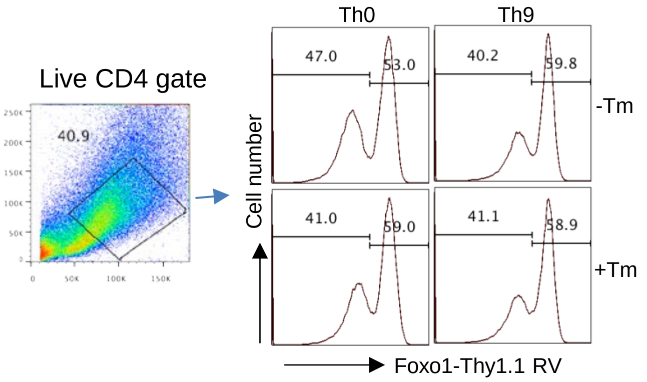

I

gating strategy for fig. 6 h

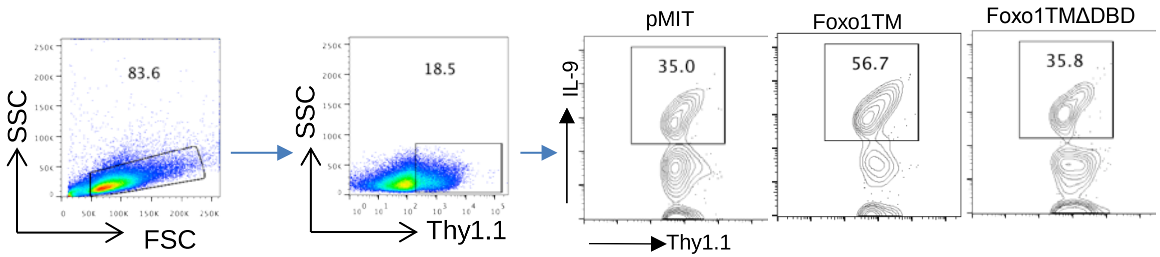

Supplementary Fig 1. **Sorting strategy of mice and human naïve $CD4^+$ T cells.**

FACS sorting strateg of naive $CD4^+$ T cell subsets from mice spleen and lymph nodes, **(a)** total splenocytes and lymph nodes cells were harvested, and then single cell suspension was prepared. Cells were gated on lymphocyte gate and size discrimination, doublet exclusion by double discrimination (FSC-H vs FSC-W and SSC-H vs SSC-W) were performed. $CD4^+CD25^-$ T cells were further sorted based on $CD62L^+$ and $CD44^-$ gate as indicated. Purity of cells were tested in post-sort fraction. **(b)** Alternatively, total splenocytes and lymph nodes cells were positively selected with anti-CD4 magnetic microbeads, and then total $CD4^+$ T cells were further sorted using anti-CD62L antibody. Purity of cells were tested in post-sort fraction. © Highly purified naive $CD4^+CD45RA^+$ T cell subsets were obtained from healthy adult PBMC. Size discrimination, doublet exclusion by double discriminations method (FSC-H vs FSC-W and SSC-H vs SSC-W) were performed before gating them on $CD4^+$ fraction, $CD4^+$ naïve T cells were further sorted based on $CD4^+CD45RA^+$ $CD45RO^-$ gate. Purity of cells were tested in post-sort fraction.

Gating strategy for various experiments (**d-i**). (**d, e**) Sorted naïve $CD4^+$ T cells were activated and transduced with either EV or AKT-DN or AKT-CA RV, cells were cultured into Th9 culture condition, $CD4^+$ cells were gated on live gate, which further gated on GFP gate as indicated. Gating in d, e correspond to figure 2 f, g of main figure. **(f)** Gating strategy for figure 3 e. Briefly activated cells were gated based on SSC and FCS, which were further gated on live cells. Live cells were then gated on $CD4^+$ T cells in which IL-9 staining was tested. **(g)** Gating strategy for figure 4 n. Briefly sorted naïve $CD4^+$ T cells were activated as indicated, cells were gated on live cells based on 7AAD staining. IL-9/IL-17 gating is done in 7AAD-$CD4^+$ cells. (**h, i**) Gating strategy for figure 6 f, h. Purified cells were gated on activated gate in which $Thy1.1^+$ cells were shown. (i) activated cells were gated based on SSC and FCS, which further gated on Thy1.1 gate. IL-9 production was shown in $CD4^+$ $Thy1.1^+$ gate.

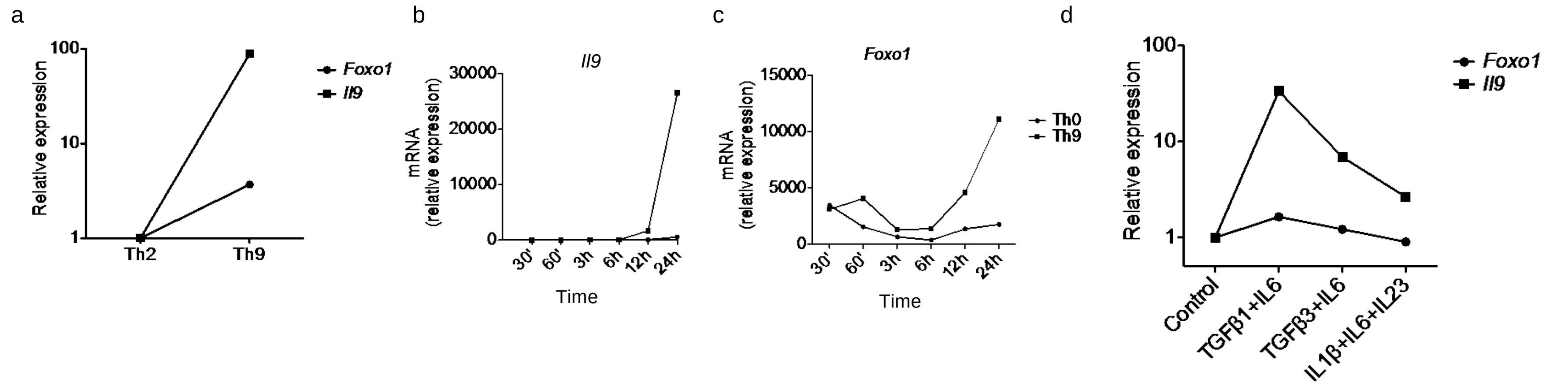

Supplementary Fig 2. Kinetic expression of IL-9 and Foxo1 in Th9 cells.

(a) Microarray samples (Th2 cells vs Th9 cells) were reanalyzed with online Geo2R software as described in methods, the values presented here representative as relative expression of *Foxo1* and *Il9* in log. **(b, c)** Sorted naïve $CD4^{+}CD62L^{+}$ T cells were differentiated into Th9 culture condition (TGF-β1 + IL-4) for different time points as indicated, mRNA expression for *Il9* and *Foxo1* by was determined by qPCR. **(d)** Microarray samples (TGF-β1 + IL-6 vs TGF-β3 + IL-6) were reanalyzed with online Geo2R software as described in methods, the values presented here representative as relative expression of *Foxo1* and *Il9* in log.

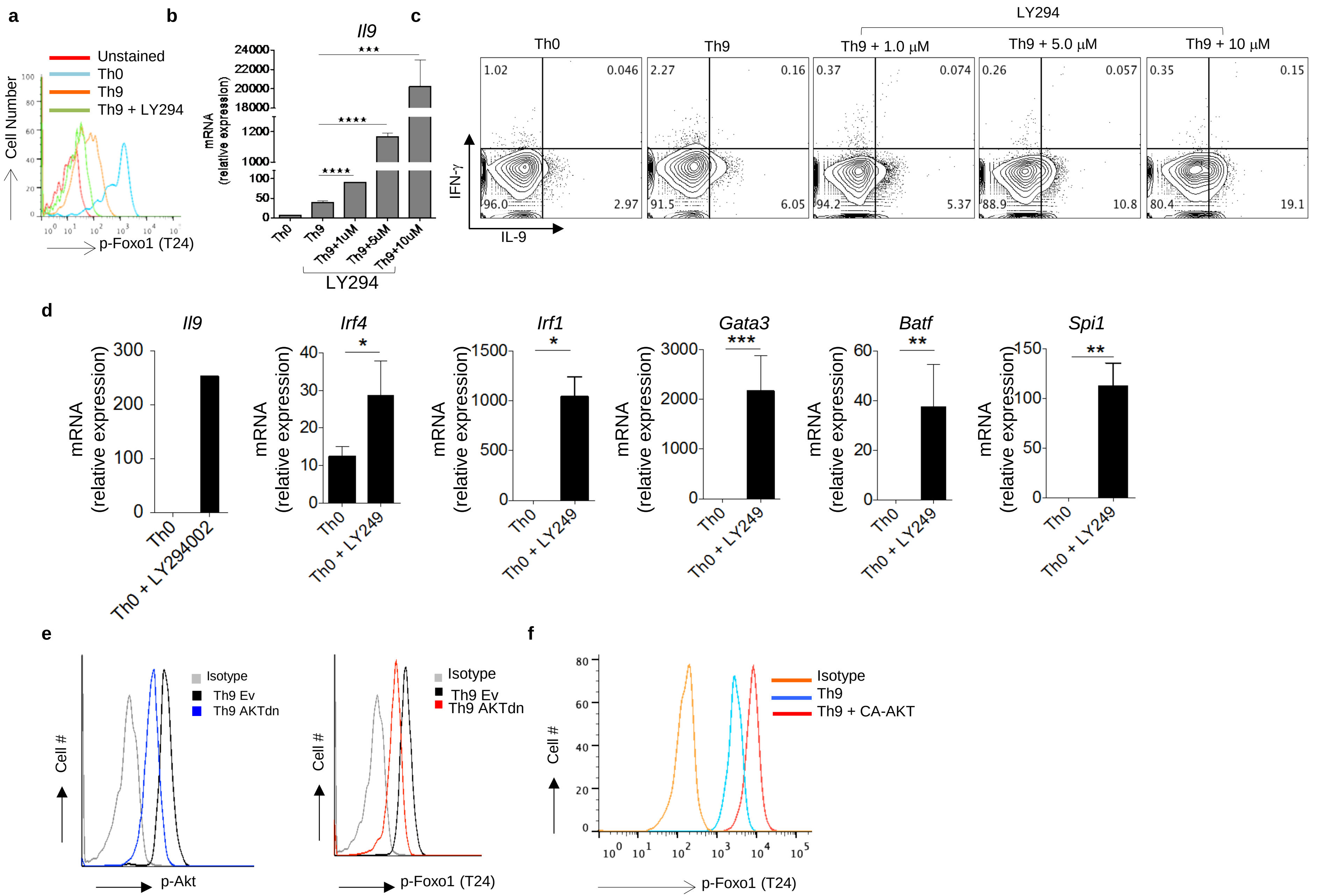
a
Unstained
Th0
Th9
Th9 + LY294
Cell Number
p-Foxo1 (T24)
b
Il9
mRNA (relative expression)
Th0
Th9
Th9+1uM
Th9+5uM
Th9+10uM
LY294
c
LY294
Th0
Th9
Th9 + 1.0 µM
Th9 + 5.0 µM
Th9 + 10 µM
IFN-γ
IL-9
1.02
0.046
96.0
2.97
2.27
0.16
91.5
6.05
0.37
0.074
94.2
5.37
0.26
0.057
88.9
10.8
0.35
0.15
80.4
19.1
d
Il9
Irf4
Irf1
Gata3
Batf
Spi1
mRNA (relative expression)
Th0
Th0 + LY294002
Th0 + LY249
e
Isotype
Th9 Ev
Th9 AKTdn
Cell #
p-Akt
p-Foxo1 (T24)
f
Isotype
Th9
Th9 + CA-AKT
Cell #
p-Foxo1 (T24)

Supplementary Fig 3. Dose dependent effect of inhibition of PI(3K)/AKT on IL-9 induction.

(a) Sorted naïve $CD4^+CD62L^+$ T cells were cultured into Th9 condition with or without LY294001, phosphorylation of Foxo1 was measured at 48 hrs. **(b)** Sorted naïve $CD4^+CD62L^+$ T cells were cultured into Th9 condition (TGF-β1 + IL-4), with or without indicated doses of LY294002, at 72 hr, RNA was extracted and qPCR was performed to determine *Il9* mRNA expression. Bars show ± s.d. of three experiments. ***P<0.001, ****P<0.0001 (Student's t-test). **(c)** On day three, cells were restimulated with PMA/Ionomycin/Golgi Stop for 4-6 hrs, intracellular production of IL-9 and IFN-γ was determined by flow cytometry. **(d)** Sorted naïve T cells were were cultured into Th9 condition with or without LY294001 (5.0 μM), mRNA expression of *Il9*, *Irf4*, *Irf1*, *Gata3*, *Batf* and *Spi1* was determined by qPCR. Bars show ± s.d. of three experiments. *P<0.05, **P<0.01, ***P<0.001 (Student's t-test). **(e)** Naïve $CD4^+$ T cells were differentiated into Th9 condition and transduced with either EV or AKTDN-RV, phosphorylation of AKT and Foxo1 was determined intracellularly by flow cytometry. **(f)** Naïve $CD4^+$ T cells were differentiated into Th9 condition and transduced with either EV or AKT-CA RV, phosphorylation Foxo1 (T24) was determined as indicated.

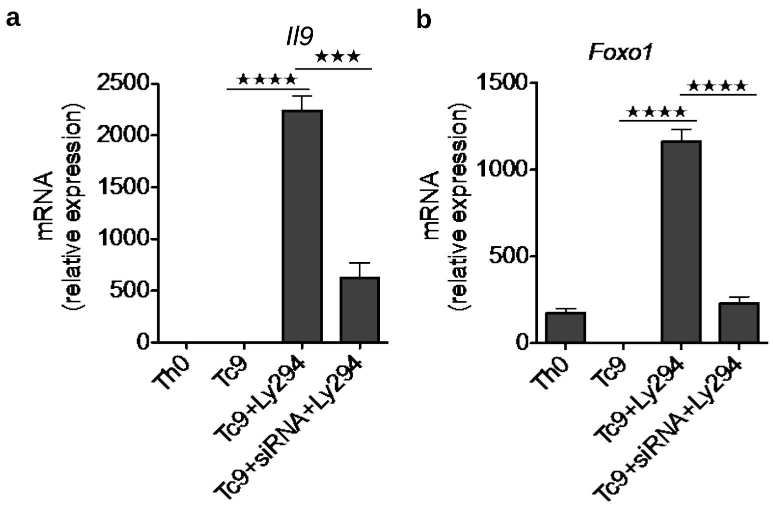

Supplementary Fig 4. Inhibition of Foxo1 suppresses the effect of LY294002 on IL-9 induction.

(a, b) Sorted naïve CD8$^+$ T cells were cultured with TGF-β + IL-4 with or without LY294002 (5.0 μM), and these cells were also treated with either Scr (sramble) or Foxo1-specific (Foxo1-siRNA) siRNA. Three days later, mRNA expression of *Il9* and *Foxo1* was determined by qPCR. Bars show $\pm$ s.d. of three experiments. *****P*<0.0001 (Student's t-test).

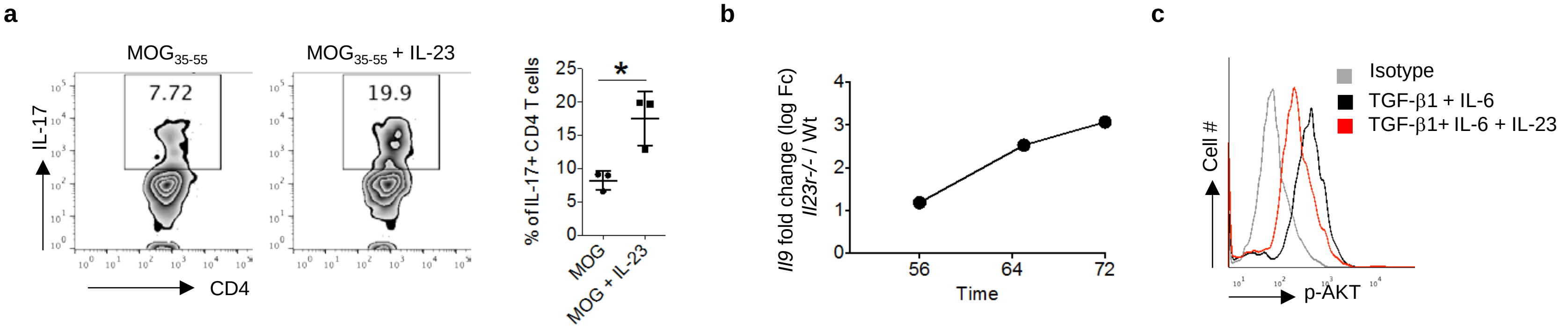

Supplementary Fig 5. IL-23 suppresses IL-9 by inducing Foxo1 phosphorylation.

(a) C57Bl/6 mice were immunized with $MOG_{35\text{-}55}$/CFA (n=3). At day 8, lymph nodes cells were re-stimulated with $MOG_{35\text{-}55}$ peptide in the presence of IL-23, percentage of $CD4^{+}IL\text{-}17^{+}$ was determined by flow cytometry. Bars show $\pm$ s.d. three experiments. *P<0.05 (Student's t-test). **(b)** The published microarray data was reanalyzed by GEO2R software and the fold change of *Il23r* was compared between Wt and *Il23r-/-* Th17 cells at indicated time points. **(c)** Sorted naïve T cells were differentiated as indicated, and then phosphorylation of AKT was determined by flow cytometry.

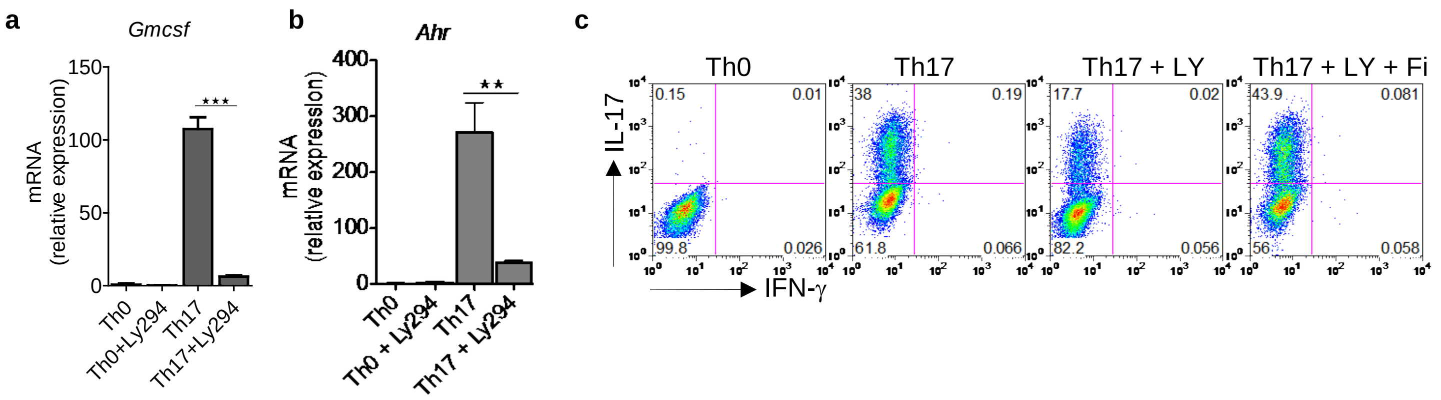

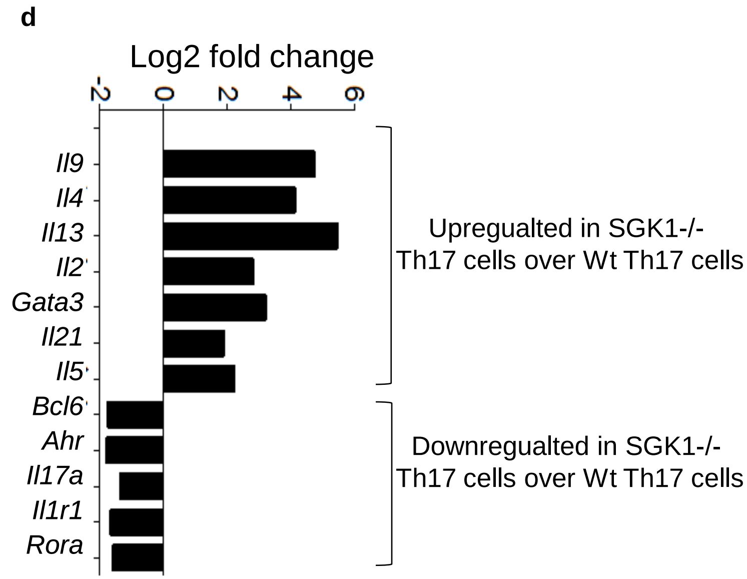

Supplementary Fig 6. Effect of LY294002 on Th17 cells associated gene.

(a-b) Sorted naïve $CD4^{+}CD62L^{+}$ T cells were cultured into Th17 condition (TGF-β1 + IL-6) with or without LY294002 (5.0 μM), at day three, mRNA expression of *Gmcsf* and *Ahr* was determined by qPCR. Bars show ± s.d. three experiments. $^{**}P<0.01$, $^{***}P<0.001$ (Student's t-test). **(c)** Sorted naïve T cells were cultured into Th17 condition with combination of LY294002 (5.0 μM) and Foxo1 inhibitor (20 nM), intracellular IL-17 and IFN-γ production was determined by flow cytometry. **(d)** The published microarray data was reanalyzed by GEO2R software and the up and down regulation of indicated genes were shown in Wt and *Sgk1-/-* Th17 cells.

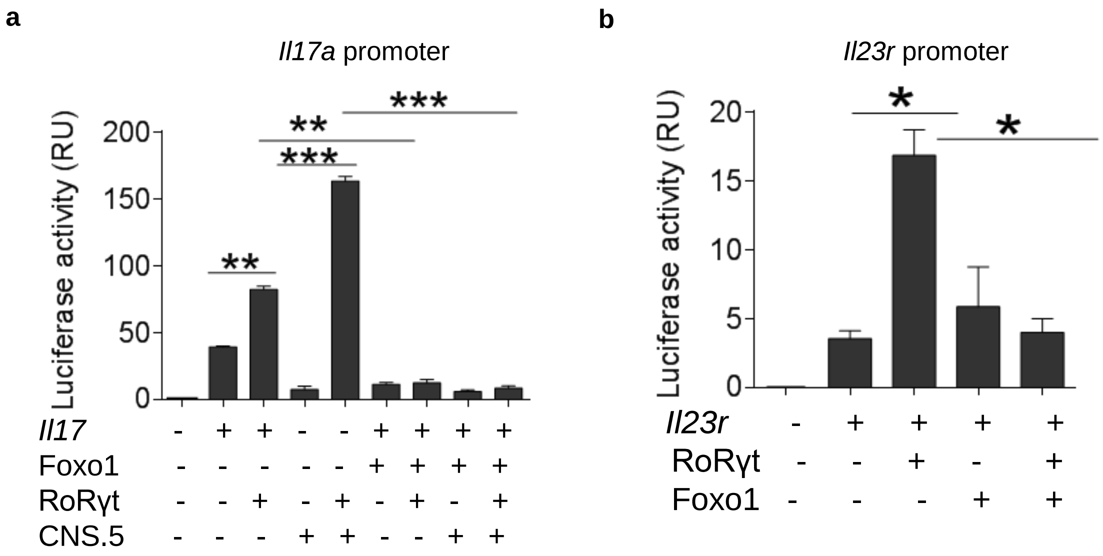

Supplementary Fig 7. Foxo1 suppresses Rorγt-induced *Il17* and *Il23r* promoter activity.

HEK-293T cells were transfected with luciferase promoter construct of *Il17A*, *Il17CNS5* and *Il23r*, cells were also co-transfected with either Wt Foxo1 or Rorγt. 24h after later, firefly and renilla luciferase activity was measured by Promega dual luciferase glow kit according to the manufacturing protocol **(a, b)**. Bars show mean ± s.d. from combined three experiments. *P<0.05, ** P<0.01, ***P<0.001 (Two tailed Student's *t*-test).

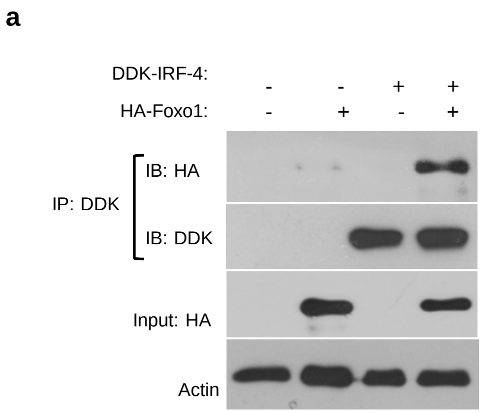

Supplementary Fig 8. Foxo1 and IRF4 interact physically.

HEK-293T cells were co-transfected with HA-Foxo1 and/or DDK-RF4, 48 hrs later cells were lysed with RIPA buffer. Protein lysate was Immunoprecipitated with anti-DDK antibody and Immunoblot was performed with ant-HA antibody **(a).**

Supplementary Figure 9

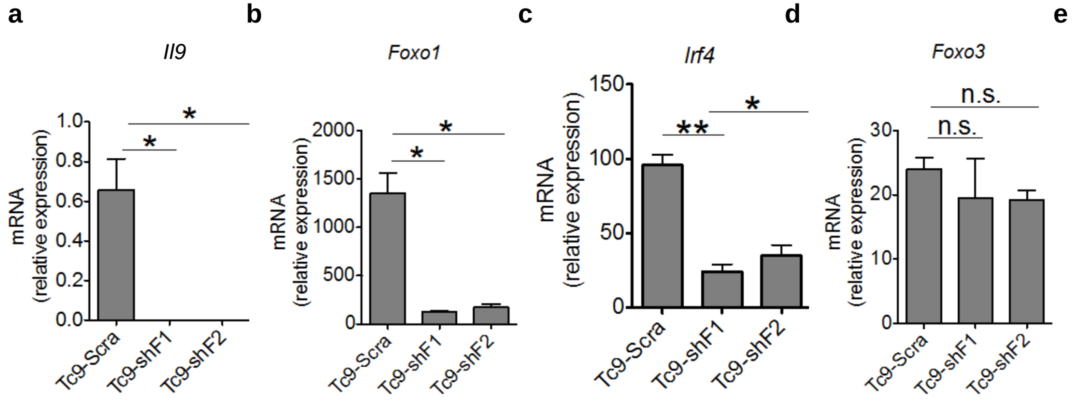

e

% inhibition	Tc9-shF1	Tc9-shF2
Il-9	-100	-100
Foxo1	-69.59	-67.39
Irf4	-90.48	-83.72
Foxo3	-40.68	-26.33

(-) Inhibition; (+) No inhibition/Activation as compared to Scrambled-ShRNA

Supplementary Fig 9. Foxo1 ShRNA suppresses IL-9 expression in Tc9 cell.

(a-d) $CD8^+CD62L^+$ T cells were sorted and transfected with either scramble (Scra) or two different Foxo1ShRNA (shF1 and shF2), transfected cells were differentiated into Th9 culture condition (TGF-β1 + IL-4) for 3-4 days. mRNA expression of *Il9*, *Foxo1*, *Irf4* and *Foxo3a* was determined by qPCR. Bars show mean ± s.d. from combined three experiments. *P<0.05, **P<0.01 (Student's *t*-test). **e)** % inhibition was calculated for indicated genes.

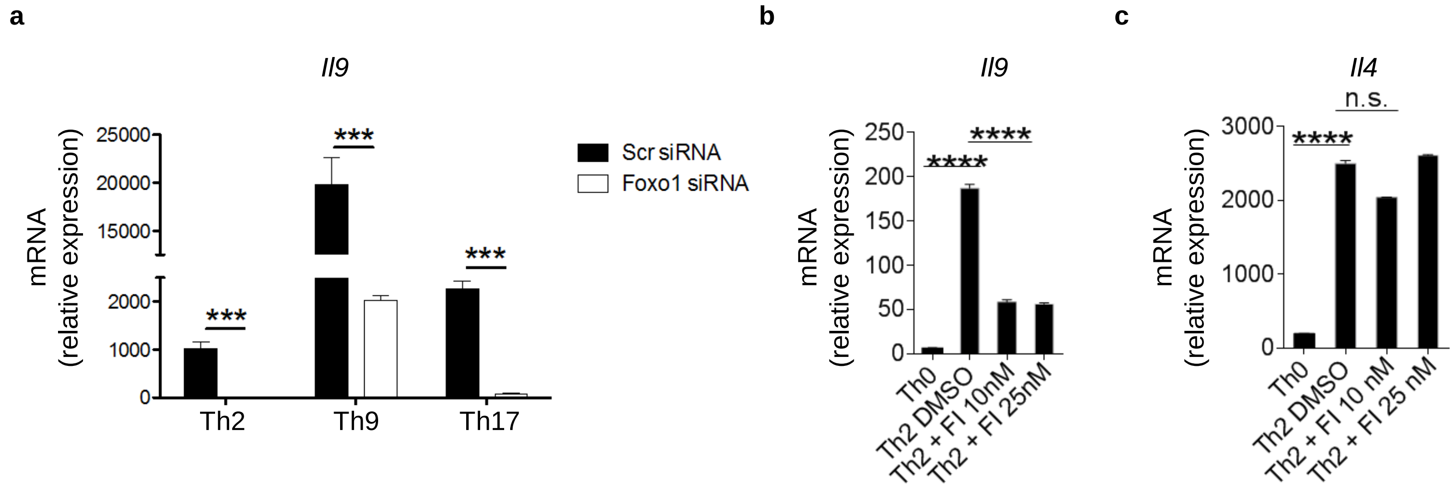

Supplementary Fig 10. Foxo1 is essential to induce IL-9 in Th2 cells.

(a, b) Naïve CD4+ T cells from Foxp3-GFP-KI mice were activated with plate bound anti-CD3 (2μg/ml) and anti-CD28 (2μg/ml) in the presence of IL-4 (10 ng/ml) plus anti-IFN-γ antibody, Taqman PCR was performed for indicated genes. Bars show mean ± s.d. from combined three experiments. ****P*<0.001, *****P*<0.0001 (Student's *t*-test).
